# Digital Stress Induction in Daily Life Using the Salzburg Mobile Stress Induction (SMSI): Development and Ambulatory Evaluation Study

**DOI:** 10.2196/75785

**Published:** 2025-09-18

**Authors:** Thomas Vikoler, Heike Ganesch, Jasmina Dubravac, Eva Traut-Mattausch

**Affiliations:** 1 Department of Psychology University of Salzburg Salzburg Austria

**Keywords:** ambulatory assessment, acute stress, negative affect, positive affect, cognitive stress test, within-person, smartphone, digital health, mobile health, open access material, mobile phone

## Abstract

**Background:**

The use of digital technology enabled examining stress in everyday life. However, ambulatory research depends on the natural occurrence of stressful situations while most standardized stress inductions rely on cost- and labor-expensive laboratory experiments, which are limited in their infrequent applicability.

**Objective:**

We developed the Salzburg Mobile Stress Induction (SMSI), a newly conceptualized toolbox including 6 different stress-inducing paradigms (Matrices test, Cube Net test, Arithmetic test, Number Series test, Word Scramble test, and Word Pair test) and 1 control paradigm (Caesar Cipher test), which are based on cognitive performance tests. These 7 tests aim to provide researchers with an open-access, standardized method to repeatedly induce stress in an ambulatory setting.

**Methods:**

We recruited university students from a local university and through a crowdsourcing platform for a preregistered ambulatory study. After completing a web-based survey, participants used the m-path app on their smartphones to conduct the 7 SMSI tests in a randomized order over 4 days. By comparing the stress-inducing tests with the control test, we investigated changes in momentary negative and positive affect from baseline (t0) to between (t1) and after (t2) each test using the International Positive and Negative Affect Schedule Short Form.

**Results:**

A total of 100 participants (60/100 women; mean age 24.43, SD 6.21 years; 69/100 local sample; 31/100 crowdsourcing sample) completed all 7 SMSI tests. Participants’ negative affect significantly increased during all 6 stress-inducing tests compared to the control test from t0 to t1 (*P*s<.001) and from t0 to t2 (*P*s<.001) with medium to large effect sizes (η_p_²s=0.10 to 0.30). Post hoc pairwise comparisons showed significant increases of negative affect during all stress-inducing tests from t0 to t1 (*P*s<.001) and from t0 to t2 (*P*s<.001) and a slight increase in the control test from t0 to t2 (*P*=.006). Reported positive affect significantly differed between the stress-inducing tests and the control test from t0 to t1 (*P*s<.001) and from t0 to t2 (*P*s<.001) with medium to large effect sizes (η_p_²s=0.14 to 0.33). Post hoc pairwise comparisons revealed a significant increase in positive affect in the control test from t0 to t1 (*P*<.001) and from t0 to t2 (*P*<.001) and varying significant decreases to nonsignificant changes in the stress-inducing tests over time (*P*s>.99 to <.001).

**Conclusions:**

The SMSI presents novel and easy-to-implement standardized stress induction procedures to repeatedly induce stress in ambulatory research. We discussed new opportunities for positive eustress inductions and outlined subsequent validation studies combining physiological stress assessment and ambulatory methods. The development of additional language versions of the SMSI is illustrated.

## Introduction

### Relevance and Rationale

Stress is a daily experience that fluctuates throughout the day and across days, weeks, months, years, and even over a lifetime. Stress impacts on health have been extensively studied: stress has been identified as a major cause of cardiovascular diseases, diabetes, dysregulation of the immune system, cognitive impairments, and mood disorders [1-3]. To better assess and manage stress in daily life, research has increasingly drifted from laboratory, cross-sectional, or continual panel studies and toward ambulatory methodological approaches that capture stress as it occurs and deliver interventions in real time. These include ecological momentary assessments [4] or ecological momentary interventions [5], respectively. However, ambulatory methods are limited in their ability to optimally address stress, as they must operate accurately when stress occurs or is likely to occur [6]. To circumvent this challenge, researchers conventionally apply stress induction paradigms in an experimental setting. These paradigms provide a reliable framework for examining acute stress responses or evaluating the effectiveness of stress management interventions.

### Current (Digital) Stress Paradigms and Their Limitations

Stress induction methods can be categorized into 3 types: physical, physiological, and psychological (see the study by Brunyé et al [7] for an overview). Most existing stress paradigms are conducted in the laboratory to facilitate standardization at the expense of their applicability in real-world situations. Most of them are time- and cost-intensive, require direct participant-investigator interactions in artificial laboratory settings, can only be repeated at long intervals, and are limited in their application to small sample sizes. To examine acute stress responses in everyday life in a standardized way, validated stress paradigms are needed, which can be integrated into a digital environment to overcome these limitations.

The Trier Social Stress Test (TSST) [8] is the most popular stress paradigm and is considered the gold standard in experimental research. The TSST procedure involves a mock job interview and an arithmetic calculation section conducted in front of an audience. Additionally, a web-based version has been developed to reduce the resource intensity and procedure variability. Both the traditional and the virtual TSST versions have been shown repeatedly to effectively induce stress (see meta-analyses by Gu et al [9] and Helminen et al [10], respectively). However, both versions of the TSST have mostly been tested in laboratories. When applied remotely, they still require live participant-investigator interactions and significantly remove participants from their everyday lives [11]. Furthermore, multiple exposures at short intervals decrease the effectiveness of the traditional and the virtual TSST, thereby indicating habituation effects [12] that limit repeated examinations of stress responses in everyday life. Although web-based stress paradigms underline the relevance of standardized examinations of acute stress responses in everyday life, they mirror laboratory experiments and do not represent ambulatory stress inductions in everyday life.

The Digital Stress Test (DST) [13] recently introduced a first prototype for remotely inducing stress without requiring standardized contact with researchers. The DST includes smartphone-based procedures of arithmetic calculations and a free speech task paired with social evaluation elements (video recording and performance comparisons). The authors reported significantly higher negative affect after completing the entire DST compared to their neutral control test (a procedure similar to the stress test, but with less stressful framings and tasks). When examining the participants’ experience of stress throughout the DST, its performance test characteristics and the arithmetic calculation task were identified as the most stressful elements. However, as the DST is a newly developed tool, insights about the effects of repeated applications are currently unavailable. When comparing similar tests such as the Montreal Imaging Stress Test [14], one study using a version for repeated application showed signs of anticipatory and thus habituation effects within the same test sessions [15]. Nonetheless, the DST demonstrates that cognitive performance tests can feasibly induce stress in everyday life. Ambulatory research can significantly benefit from the daily stress inductions by circumventing stressor occurrences by chance [6] and enabling standardized targeted investigations of stress responses and reactivity [16]. To address this issue, we provide a toolbox of multiple newly developed cognitive performance tests that operate on a standardized digital procedure.

### Salzburg Mobile Stress Induction

#### Overview

We introduce the Salzburg Mobile Stress Induction (SMSI), a newly conceptualized and developed toolbox that enables researchers to remotely induce stress in everyday life. The SMSI comprises various distinct stress paradigms that repeatedly evoke acute stress responses in the participants’ familiar environment while minimizing habituation effects [12]. Additionally, the SMSI can operate solely digitally without requiring direct communication between the participant and the research coordinator—from installation (eg, web browser–based) to execution (eg, app-based via m-Path [17]).

#### Concept of the SMSI Tests

**Figure 1:**
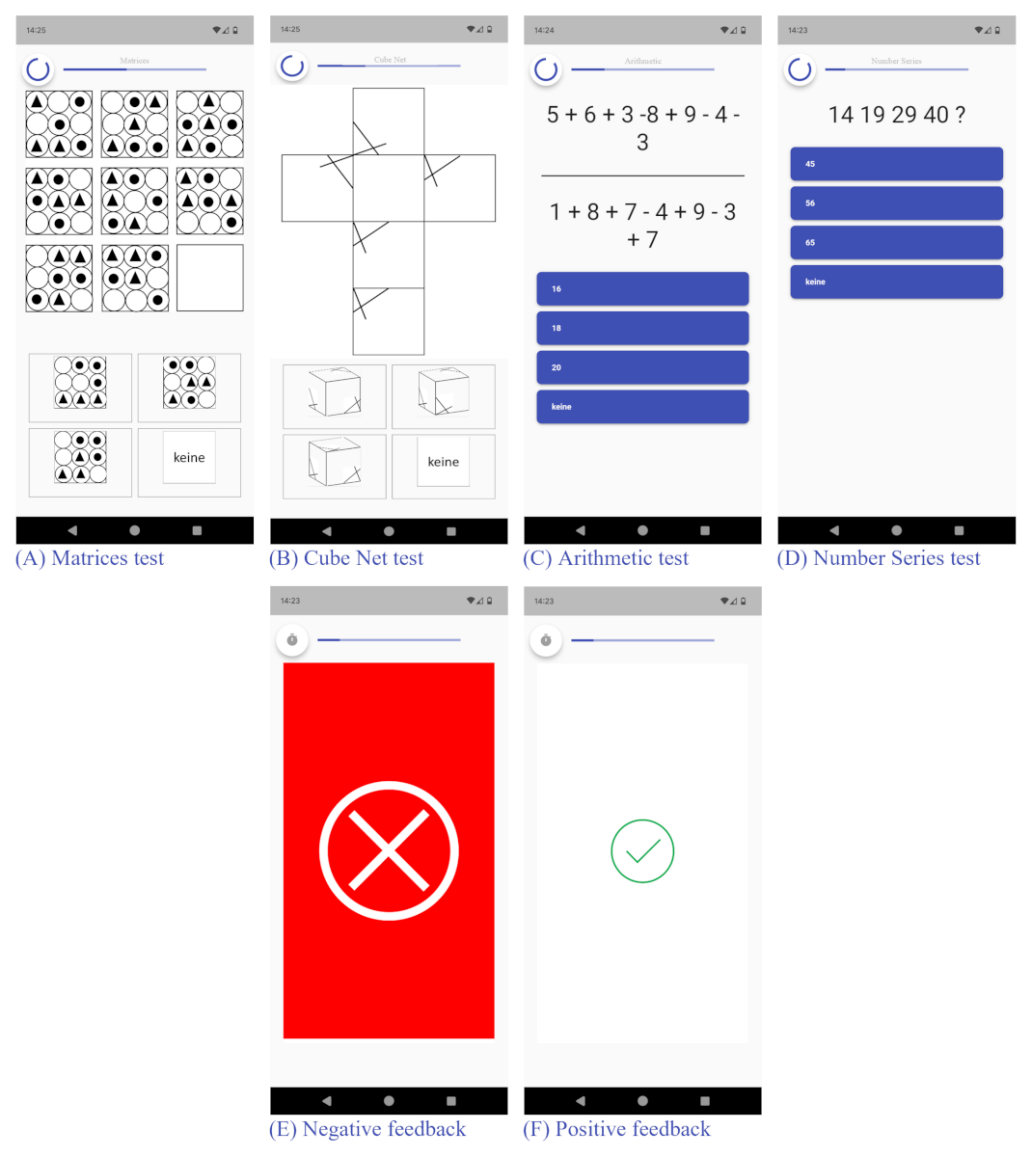
Example screenshots from the Salzburg Mobile Stress Induction. Top row—Salzburg Mobile Stress Induction tests: (A) Matrices test, (B) Cube Net test, (C) Arithmetic test, and (D) Number Series test. Bottom row—response feedback: (E) negative feedback, and (F) positive feedback.

The SMSI comprises 7 different cognitive tests—6 stress-inducing tests and 1 neutral control test. All 7 tests are presented as cognitive intelligence tests for application in everyday life. Alongside a variety of tests, we intended to ensure that these also differed in terms of content. Two of the 6 stress-inducing tests each comprise figural (Matrices test [MT] and Cube Net test [CN]), numeric (Arithmetic test [AR] and Number Series test [NS]), and word-based tasks (Word Scramble test [WS] and Word Pair test [WP]), while the seventh neutral control test (Caesar Cipher test [CC]) was designed as a word-based test as well. The 3 word-based tests we present in this study are in the German language. An English language version of the test materials has already been developed and is currently undergoing validation (T Vikoler, G, Biczkowski, L Brandt, M Brunner, E Traut-Mattausch, unpublished data, 2025). Figure 1 and Figures S1-S4 in Multimedia Appendix 1 display example screenshots of the SMSI test tasks and applied feedback. The shared layout of all 7 tests is described below. The sections below detail the varying characteristics of the individual stress induction tests and the neutral control test. Each test comprises 2-3 blocks with 12 single-choice tasks each. To answer the single-choice questions, 4 options were presented, with the fourth indicating that none of the 3 answers were correct. Participants had a constant time limit of 12 seconds to respond to each question, except in the WP, which includes a 1-minute memory phase followed by a 5-second response time per question. After each question, immediate feedback: incorrect answers or late responses induce prominent negative feedback that is displayed in the form of a bright red screen with a large white cross at the center (Figure 1E). In contrast, correct answers display a small green tick on a white background (Figure 1F).

#### About MT

The MT paradigm is based on commonly used intelligence matrix tests (eg, Raven Progressive Matrices [18]) and is presented as a test of perceptual and logical thinking skills. Each task comprises a 3×3 grid filled with figures and shapes, alongside a blank field (Figure 1A). The objective is to determine which figure logically completes the pattern based on the structural rules of the matrix. These rules may apply either horizontally, vertically, diagonally, or in a combination thereof within the matrix. As difficulty increases, the designs increasingly get elaborate and intricate while the logical rules increase in complexity (eg, rotation rules with different rotation degrees). Furthermore, conditional and other rules were introduced (eg, the presence or absence of patterns in one field determines the shading or position within the field of a preceding element) to enhance the difficulty level. In addition to the increasingly difficult rules and patterns in the depicted matrix and the answer options, the position of the blank fields within the matrix changes throughout the test, thereby increasing unpredictability.

#### About CN

The CN paradigm is based on spatial ability tests (eg, the study by Pawlak-Jakubowska and Terczyńska [19]) and it evaluates spatial imagination and logical thinking skills. In this test, a 2D cube net of a 3D cube is presented to participants to determine which of the given 3D cubes could be constructed by folding the given net (Figure 1B). The cube nets include varying patterns and forms (eg, geometrical forms or shadings) in varying positions. With increasing difficulty, the designs on the cubes’ sides become more detailed, including more complex asymmetrical patterns or even identical sides. These configurations heighten the cognitive processing and challenge the spatial visualization ability by requiring more mental rotations of the cubes.

#### About AR

The AR paradigm is a test used to assess mathematical skills. Each question displays 2 equations involving the addition and subtraction of numbers between 1 and 9, separated by a horizontal line (Figure 1C). The objective is to solve each equation separately and then subtract the smaller result from the larger one. Between the 2 equations, the equation yielding the higher results varies throughout the test. The answer options displayed range from single- to double-digits. As the difficulty increases, the number of summands and subtrahends in the arithmetic equations increases progressively from 2 to 6 operators. The simultaneous calculation of up to 14 numbers in 2 equations and the memorization of the intermediate results contribute to the overall cognitive load.

#### About NS

The NS paradigm is based on common intelligence tests (eg, part of the intelligence structure test 2000 R [20]) and conducts numerical sequences. In this test, various numbers are presented to identify the sequence rule and determine the next number (Figure 1D). The array of numbers ranges from 4 to 7 figures, including single-, double-, and triple-digit numbers. The tasks apply different principles: additions, subtractions, multiplications, prime numbers, cross sums, exponentiates, and Fibonacci sequences. As the difficulty progresses, the complexity of the applied principles increases and the principles become interwoven (eg, subtract prime numbers) to challenge the cognitive processing and pattern recognition ability.

#### About WS

The WS paradigm is designed to assess verbal reasoning and word recognition abilities. The WS is the first of 2 tests of the SMSI, which is based on a pseudointelligence test. The public’s perception that WS tasks are equivalent to intelligence tests is demonstrated by the 2600 and 102,000 Google search results yielded for the essential keywords “word scramble” and “intelligence test” or “IQ,” respectively. The test aims to unravel the displayed words with intermingled letters and identify the correct first letter of the sought word (Figure S1 in Multimedia Appendix 1). As difficulty increases, word length increases from 4 to 13 letters. Additionally, difficulty increases by incorporating more complex words, including repetition and double occurrences of vowels or consonants. Moreover, more specialized and technical vocabulary is introduced to further enhance the challenge of word recognition.

#### About WP

The WP paradigm tests the memory capacity and is based on widely used instruments for assessing explicit episodic memory, such as the Verbal Paired Associates test (subtest of the Wechsler Memory Scale [21]). Here, participants are given 1 minute to memorize a list of word pairs (Figure S2 in Multimedia Appendix 1). Afterward, any first word from one of the previously presented word pairs is displayed to select the corresponding matching word (Figure S3 in Multimedia Appendix 1). After 6 questions, a new word pair list of 4 is introduced. To align WP duration with other SMSI tests due to the memorization phases, and to increase the level of difficulty, the time to answer each question was shortened (5 seconds). The difficulty level increases over time through various schedules: first, the length of the word pair lists increases while the memorization time remains the same. Second, the used words become more complex and technical, and less familiar terms are chosen (eg, concrete versus abstract words), requiring more intricate and elaborate associations between the paired words. Third, as word pairs with or without similar prefixes or alliterations and word pairs with and without semantic relationships are used, the difficulty level increases with the structural level of the memorized and questioned word pairs. Fourth, words from both previous word pair lists and questioned words are incorporated in subsequent word pair lists as well as questions to increase interference effects and to decrease the ability to distinguish and recall the correct second words.

#### About CC—Neutral Control Test

The CC paradigm tests text-encrypting ability and serves as a neutral, nonstress-inducing control test for comparing the stress-inducing tests of the SMSI. The CC is the second SMSI test that is not based on scientifically based intelligence tests, but on a well-known encryption technique known as the Caesar cipher technique. word scrambles, encryption tests are commonly perceived as an intelligence test equivalent, yielding 34,900 Google search prompts for the keywords “encrypt” and “intelligence test,” or 331,000 prompts for “encrypt” and “IQ.” The general CC design mirrored the experimental tests, including the procedure, design, number of questions, and time limit. However, the difficulty level remains within a moderate solvable level by applying a straightforward rule-based approach. Contrary to famous digital-cipher tasks—in which the objective is not to decipher words with interchanged letters—our test requires participants to encrypt a given word using the Caesar cipher encryption code—by moving each letter down the alphabet by a set number of positions (eg, with an encryption code of 2 the letter “A” changes into a “C” or the letter “B” changes into “D”; see Figure S4 in Multimedia Appendix 1). By minimizing the mental steps required to obtain the correct response, the low difficulty compared to the stress-inducing paradigms is achieved by adapting the response options. A maximum of 2 letters separated the presented potential decrypted words, making them quite familiar. Instead of moving every letter of the word down the alphabet, this intentional design enables easy-to-apply strategies of focusing only on the varying parts of the response options and the corresponding letters of the original word. With easier tasks, the time limit is consequently less demanding, thereby resulting in better results with less arousing negative feedback. However, to maintain the appearance of a performance-based intelligence test, an increase in difficulty was mandatory. This was achieved by gradually lengthening the words to be decoded, from 3 letters up to 6 letters.

#### SMSI Test Material Availability

The SMSI presents a new tool for researchers to implement stress-induction paradigms in their research, be it in laboratories or ambulatory studies. To enable easy use of the SMSI following the principles of open access, the entire test materials of the SMSI presented in this study are publicly available at the Open Science Framework [22]. Additionally, we publicly shared preset structures for the immediate test import in m-Path [17] (search keywords “SMSI” or “Salzburg Mobile Stress Induction”). Nonvalidated test materials of the English version of the SMSI are available at the same location.

#### Development and Procedure of the SMSI

Building on results of the DST [13]—which identified participation in performance tests, time constraints, uncontrollability, immediate feedback, and task difficulty as one of the most stress-inducing elements—we developed 7 independent tests designed as quasi-intelligence test tasks. When developing the SMSI, we used various sources to conceptualize varying stress-inducing types of performance tests. The AR was directly influenced by the arithmetic subtest of the DST and further adapted to match the structure of the other tests. Commonly used types of intelligence tests were adduced in the development of most SMSI tests (eg, matrices for MT). Other task types were derived from internet research on cognitive tasks (eg, WS). In addition to investigating and reviewing potential stress-inducing tasks, we sought a cognitive performance task with minimal difficulty to serve as a neutral control test without being identified as such.

**Figure 2:**
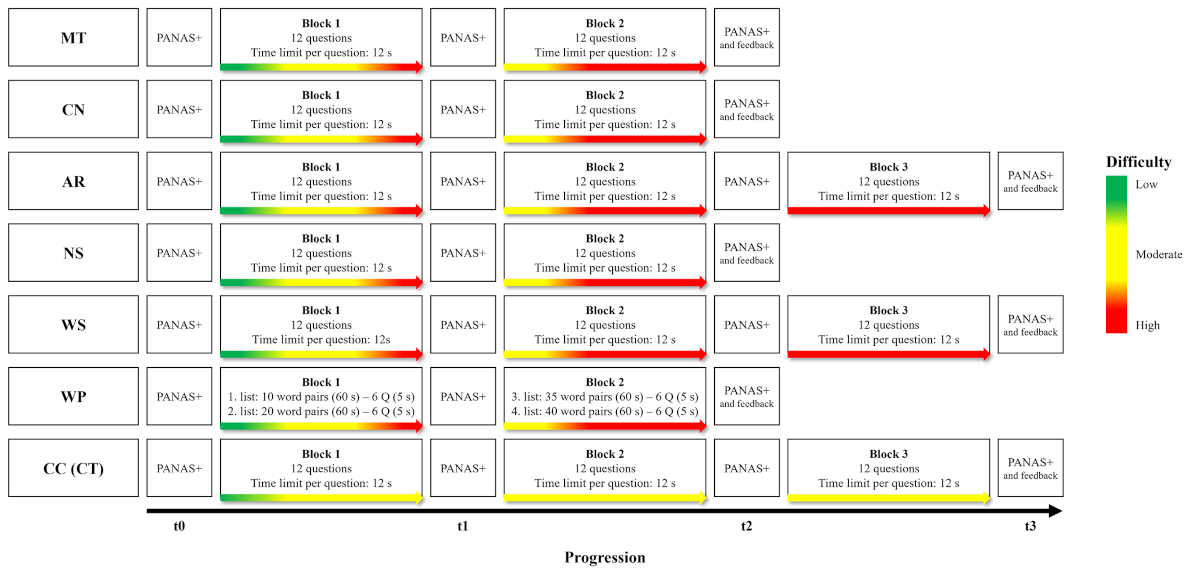
Structure of the Salzburg Mobile Stress Induction tests and assessment procedure. Feedback is the concluding feedback questions on the completed test. AR: Arithmetic test; CC (CT): Caesar Cipher test (control test); CN: Cube Net test; MT: Matrices test; NS: Number Series test; PANAS+: momentary affective assessment: 10 items of the International Positive and Negative Affect Schedule Short Form [23] plus 1 single item each on stress and frustration; Q: question; t0: baseline assessment before the first task block; t1: assessment after the first task block; t2: assessment after the second task block; t3: assessment after the third task block; WP: Word Pair test; WS: Word Scramble test.

Each test starts off with a brief introduction and 1 example item to ensure participants’ understanding of the task’s objective. Difficulty in the stress-inducing tests increased rapidly during the first block: the first 4 questions were relatively easy to solve, followed by 4 moderate to difficult questions and 4 highly difficult questions at the end of the first block. In the second block of the stress-inducing tests, only the first 4 questions were medium to difficult, whereas the remaining questions remained highly difficult. SMSI tests with 3 blocks maintained the difficulty level of the end of the second block. Contrarily, the control test only increases the questions’ difficulty to a mediocre level across all blocks. The difficulty was complemented by a time restriction for each question. Three of the 7 SMSI tests (AR, WS, and CC) included 3 instead of 2 blocks of tasks. When administering multiple or all SMSI tests repeatedly within a short duration, the different number of task blocks creates a certain feeling of unpredictability at the end of the tasks. Figure 2 provides a structural overview of the SMSI tests. After each task, we incorporated enhanced negative feedback on incorrect or overdue answers as well as simpler and negligible positive feedback on correct answers, which amplifies the feeling of failure and consequently the experience of stress. The respective feedback is displayed for only 1 second before the next question appears. Multimedia Appendix 2 exemplifies a complete smartphone procedure of the AR of the SMSI.

#### Objectives and Hypotheses

This study aimed to validate the SMSI as a toolbox for repeatedly inducing stress digitally in everyday life. Furthermore, we intended to evaluate the SMSI’s feasibility and stress-inducing potential against a neutral control test based on the same procedural principles as the other cognitive performance tests. We preregistered this study at the Open Science Framework [24] where we hypothesized that, negative affect scores increase significantly more in the experimental tests (MT, CN, NS, AR, WS, and WP) compared to the control test (CC) from baseline (before the first task block t0) to after the first task block (t1) and to after the second task block (t2; Figure 2). In addition to assessing the changes in negative affect, we also examined the effect of the 7 SMSI tests on the participants’ positive affect responses.

## Methods

### Pilot Study

We conducted a pilot study with 3 participants who performed all 7 tests over 4 days to test the basic operability of the SMSI’s smartphone procedure in conjunction with the used m-Path software [17]. We adjusted usability issues and revised for comprehensive m-path installation and test introductions.

### Evaluation of the SMSI

#### Overview

To examine the feasibility and stress-inducing potential of the SMSI, we first conducted a longitudinal web- and smartphone-based study. In this study, participants conducted all 7 SMSI tests while surveying their affective states.

#### Participants and Recruitment

##### Overview

**Figure 3:**
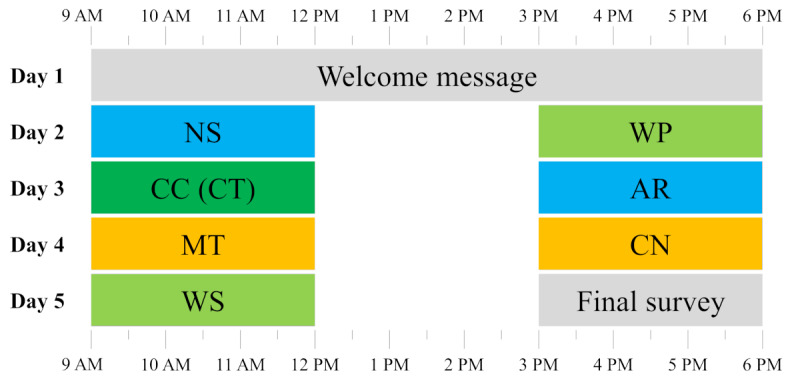
Exemplary schedule of the SMSI smartphone study procedure. Colored backgrounds indicate different sections or SMSI test types: grey: subsidiary parts (welcome message: notification about the pending start of the study and final survey: feedback questions), blue: numeric tests, green: word-based tests (dark green: control test), and orange: figural tests. Tests were randomized between participants. AR: Arithmetic test; CC (CT): Caesar Cipher test (control test); CN: Cube Net test; MT: Matrices test; NS: Number Series test; SMSI: Salzburg Mobile Stress Induction; WP: Word Pair test; WS: Word Scramble test.

Participants consisted of only university students and were recruited in 2 ways: first, by distributing the link of the web-based initial survey within the local university network via social networks (eg, Facebook and WhatsApp), university e-mail lists, and in university courses (via QR code and mail lists). Second, by distributing the link of another web-based initial survey (see Framing and Incentive section) via crowdsourcing. A total of 229 participants from the local university subsample (164/229, 71.6%) and the crowdsourcing subsample (65/229, 28.4%) completed the initial survey, registered in m-Path [17], and were eligible for the smartphone study procedure. The 7 SMSI tests were randomized within each participant and completed over 4 consecutive days (2 tests per day; Figure 3). In the end, 129 (56.3%) of the 229 participants did not complete all SMSI assessments and were consequently excluded from the analysis.

##### Framing and Incentive

This study was framed as “a study about the validation of a new smartphone-based tool for testing cognitive intelligence in everyday life via numerous cognitive tests.” While bachelor psychology students at the local University of Salzburg were incentivized with university participation credits, crowdsourcing participants were remunerated for participating in the initial survey and taking each test. In the crowdsourcing subsample, the initial survey was framed differently—as a “study about personality, self-perception, and smartphone usage during their studies”—due to incompatible payment procedures of both the initial survey part and the smartphone study part. At the end of this initial survey, the crowdsourcing participants were invited to participate in the subsequent study, which continued with the same intelligence test cover story. During the smartphone procedure, each SMSI test was introduced separately at the beginning to test an explicit skill (eg, mathematical, spatial imagination, or logical thinking skills).

#### Study Procedure

##### Overview

The entire study procedure spanned 6 to 7 days. Participants completed the initial survey in a web browser, which included the cover story (see Framing and Incentive section) and information about this study’s procedure, informed consent, demographic questions, and trait questionnaires. At the end of the initial survey, participants received a step-by-step image-based instruction (Multimedia Appendix 3) on how to download the m-Path app on their smartphone device, register, and connect to the research account. After a day or 2, the m-Path app launched the smartphone study procedure (based on the participant’s registration time and when the investigators implemented this study using the m-Path app).

The app-based study procedure lasted for 5 days. On the first day, participants received 2 notifications on their smartphones (between 9 AM and 6 PM), reminding them that this study would begin the following morning. On the following 4 days, participants received 2 notifications per day (at 9 AM and 3 PM) to participate in an intelligence test. Each test could only be started once and was open for 3 hours. The tests were randomized within the participants using block randomization. On the afternoon of the fifth day, instead of completing a test, participants were asked to answer feedback questions about the previously completed tests, marking the conclusion of this study. Figure 3 presents an exemplary schedule of the smartphone-based study procedure.

Participants from the local university subsample were debriefed after completing the data collection. Participants from the crowdsourcing subsample were linked at the end of the final survey to an external survey regarding their payment information, and were debriefed afterward. All participants could quit this study at any time by closing (and uninstalling) the app.

##### m-Path App-Based Study Data Collection

The app-based data collection started on the second day of the smartphone study procedure (Figure 3). Each test procedure started with a short introduction, followed by the respective test instructions and a simple example task, supplemented by a detailed description of how to solve the example task. We then assessed the baseline (t0) of the participants’ momentary negative (5 items; Cronbach α=0.77 to 0.88) and positive affect (5 items; Cronbach α=0.87 to 0.93) with the International Positive and Negative Affect Schedule Short Form (PANAS [23]). The PANAS is a well-tested, reliable, and validated measure to assess positive and negative mood—which are commonly applied in ecological momentary assessment studies—especially when both negative and positive affect are simultaneously considered [25]. Table S1 in Multimedia Appendix 4 presents the detailed Cronbach α of both subscales for each test and measurement time. Additionally, 2 more items were simultaneously presented regarding the participants’ feelings of stress and frustration. These questions were rated on a visual analog scale ranging from 0 (not at all) to 100 (extremely), which showed high consistency and accuracy in ecological momentary assessment studies [26]. Afterward, the first block of the respective SMSI test started. After completing the first block, momentary affect was measured again (t1), similar to baseline. The next was the second block of tasks, followed by a third assessment of momentary affect (t2). In the CC, AR, and WS, a third block of tasks and a fourth assessment of momentary affect were conducted. Each SMSI test concluded with feedback questions regarding the performed test (eg, comprehensible instructions, difficulty of the tasks, distraction during the tasks, and more or less time required). Figure 2 shows a detailed design of the app-based study procedure and data collection.

### Statistical Analysis

#### Power

In their study, Norden et al [13] showed that the mathematics test of the DST had a group × time interaction effect of η_p_²=0.05. An effect size of *f*=0.23 was obtained by using this score within G*Power (version 3.1.9.7; Heinrich-Heine-Universität Düsseldorf). In an a priori sample size calculation for a repeated measures ANOVA with 7 groups (SMSI tests) and 3 measurements (time), the effect size was therefore set to *f*=0.23, the α error level was set to *P*=.01, and the power was set to 99% to allow correction for multiple testing and Greenhouse-Geisser adjustments. The analysis revealed a minimum required sample size of 91. In the end, we gathered data from 100 participants who conducted all 7 tests of the SMSI.

#### Statistical Analysis

Using SPSS (version 29.0.0.0; IBM Corp), two 2-way repeated measures ANOVAs were conducted to analyze the participants’ change in negative and positive affect mean scores of the PANAS [23] using the within-factors *test* (SMSI stress-inducing MT, CN, NS, AR, WS, and WP against the neutral control test CC) and *time* (t0 against t1 and t2). Contrasts were calculated for *test* (stress-inducing MT, CN, NS, AR, WS, and WP against the neutral control test CC) and for *time* (t0 vs t1 and t0 vs t2).

Given the large sample size in our study and ANOVA’s robustness against nonnormal distributed data and violations of sphericity [27], we conducted the analyses as scheduled using the recommended Greenhouse-Geisser corrected degrees of freedom for all models. To separately test the change in affect scores of each SMSI test, we conducted Bonferroni-adjusted post hoc pairwise comparisons. Additionally, we calculated separate models to control for potential confounding effects of the 2 different sampling procedures (local university vs crowdsourcing *sample*) as well as for potential *gender* differences and effects of *employment* status.

### Ethical Considerations

This study was approved by the ethics committee of the University of Salzburg (EK-GZ: 34/2014), including all aspects of this study, such as study procedures, informed consent, data processing, debriefing, and withdrawal from this study, which were conducted per General Data Protection Regulation. This study’s m-Path data processing complies with the General Data Protection Regulation. At the beginning of this study, participants were informed about the content and procedure of this study, their rights and obligations, and data processing. Participants could withdraw from this study and their consent at any time, and manually block the research account and delete their online data in the m-Path app. Informed consent was obtained from all participants at the beginning of this study (once for local students and twice for crowdsourcing students for each part of this study—see Framing and Incentive section). Personal data was collected and stored internally only, then coded and anonymized, before being deleted after this study. Participants were compensated at the end of this study (see Framing and Incentive section).

## Results

### Participants and Completion Rate

In total, 229 university students registered for the study in m-Path with their smartphones, of which 100 (43.7%) participants completed all 7 SMSI tests (69/100, local university subsample; 31/100, crowdsourcing subsample). Table 1 provides a detailed description of the compliance frequencies and ratios from study registration to completion for the total and both subsamples. Participants who dropped out of this study or missed a test did not differ significantly from participants who completed all 7 tests regarding sociodemographic or personality characteristics, or perceived stress measured at the initial survey (Table S1 in Multimedia Appendix 5). For all SMSI tests, there was a 0% (0/229) dropout rate during the test procedures, which implies that every participant who started a test and participated in a baseline assessment also completed the respective test and each subsequent assessment. Overall, the sample comprised 60 women, 34 men, and 1 person who identified as nonbinary. Demographic data from 5 participants could not be assigned to smartphone data due to a mismatch between the individually generated participant codes in the initial survey and the m-Path registration. The participants were on average aged 24.43 (SD 6.21) years. Table S1 in Multimedia Appendix 6 provides a thorough explanation of the sociodemographic information for the total sample and split for both subsamples.

**Table 1:** Compliance frequencies and rates in the total sample and both subsamples. Percentages refer to the number of participants registered for this study in the m-Path app in each subsample.

Number of completed tests	Total sample, n/N (%)	Local university subsample, n/N (%)	Crowdsourcing subsample, n/N (%)
0^a^	229 (100)^b^	164 (100)^b^	65 (100)^b^
1	206/229 (90)	153/164 (93.3)	53/65 (81.5)
2	200/229 (87.3)	149/164 (90.9)	51/65 (78.5)
3	192/229 (83.8)	144/164 (87.8)	48/65 (73.8)
4	182/229 (79.5)	135/164 (82.3)	47/65 (72.3)
5	169/229 (73.8)	125/164 (76.2)	44/65 (67.7)
6	147/229 (64.2)	105/164 (64)	42/65 (64.6)
7	100/229 (43.7)	69/164 (42.1)	31/65 (47.7)

^a^Participants who registered for this study in the m-Path app.

^b^n (%).

On average, the participants completed each test procedure in 11.34 (SD 7.54) minutes. Table 2 provides a detailed description of the required times for each test.

**Table 2:** Mean and SD of the cumulative and average completion times (in minutes) for the Salzburg Mobiles Stress Induction’s smartphone procedure and each segment, reported for the total sample and separately for the local university and crowdsourcing subsamples.

Completion times		Total sample (N=100), mean (SD)	Local university subsample (n=69), mean (SD)	Crowdsourcing subsample (n=31), mean (SD)
**Overall SMSI^a^ procedure (in minutes)**				
	Entire study procedure (sum)^b^	108.13 (71.88)	100.15 (60.24)	125.89 (91.39)
	Entire study procedure (mean)^b^	12.21 (7.96)	11.28 (6.70)	14.29 (10.04)
	Smartphone procedure (sum)^c^	92.13 (68.00)	84.12 (54.99)	109.94 (89.06)
	Smartphone procedure (mean)^c^	11.68 (8.49)	10.63 (6.88)	14.01 (11.06)
	Test procedure (sum)^d^	79.40 (52.79)	75.95 (53.71)	87.07 (50.68)
	Test procedure (mean)^d^	11.34 (7.54)	10.85 (7.67)	12.44 (7.24)
**Segments of SMSI procedure (in minutes)**				
	Initial survey^e^	16.85 (10.84)	16.76 (12.14)	17.04 (7.24)
	Matrices test	14.02 (26.10)	14.36 (30.77)	13.29 (10.05)
	Cube Net test	11.78 (11.80)	11.81 (13.48)	11.72 (6.91)
	Arithmetic test	10.95 (11.00)	9.11 (4.85)	15.05 (17.92)
	Number Series test	7.42 (5.06)	6.54 (2.71)	9.37 (7.89)
	Word Scramble test	10.20 (10.35)	8.63 (6.30)	13.68 (15.67)
	Word Pair test	10.37 (9.92)	10.48 (6.03)	10.14 (15.56)
	Caesar Cipher test (control test)	14.65 (23.21)	15.02 (26.49)	13.82 (16.62)
	Final survey^f^	14.47 (45.36)	8.95 (12.02)	28.37 (82.48)

^a^SMSI: Salzburg Mobile Stress Induction.

^b^Complete study procedure includes the initial survey, all 7 Salzburg Mobile Stress Induction tests, and the final survey.

^c^Smartphone procedure includes all 7 Salzburg Mobile Stress Induction tests and the final survey.

^d^Test procedure includes only the 7 Salzburg Mobile Stress Induction tests (including assessments).

^e^N_Total sample_=95; N_Local university subsample_=66; N_Crowdsourcing subsample_=29.

^f^N_Total sample_=88; N_Local university subsample_=63; N_Crowdsourcing subsample_=25.

### Negative Affect

**Figure 4:**
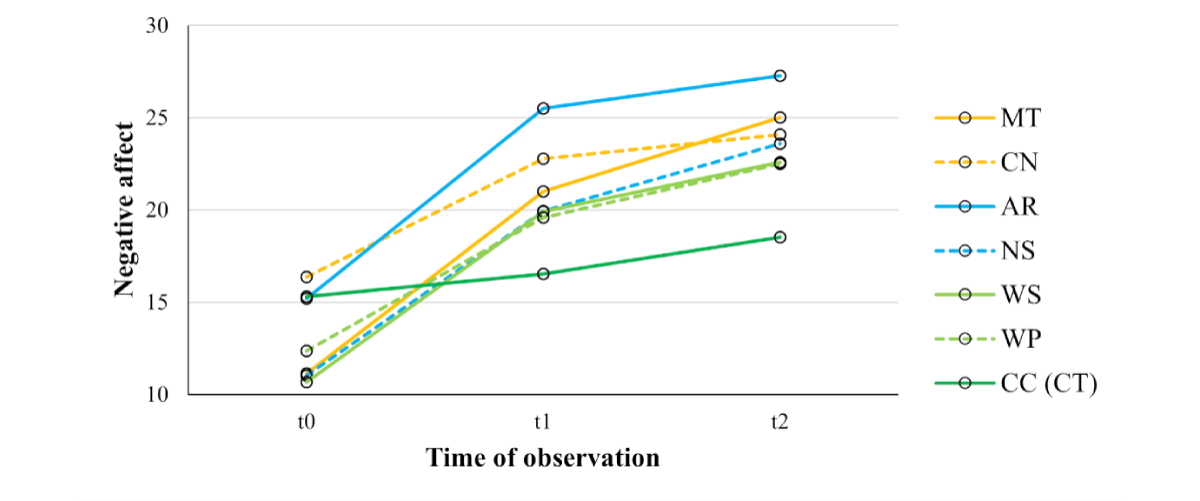
Negative affect was measured by the PANAS for each test of the SMSI across all participants (N=100) at t0, t1, and t2. Orange lines represent the figural tests (MT and CN), turquoise lines represent numerical tests (AR and NS), light green lines represent word-based tests (WS and WP), and the dark green line represents the word-based control test (CC (CT)). Negative affect scores significantly increased from t0 to t1 and from t0 to t2 in all tests except the CC (CT), where there was only a significant increase from t0 to t2. The CN and AR did not significantly increase negative affect from t1 to t2, whereas the other tests did. AR: Arithmetic test; CC (CT): Caesar Cipher test (control test); CN: Cube Net test; MT: Matrices test; NS: Number Series test; PANAS: Positive and Negative Affect Schedule; SMSI: Salzburg Mobile Stress Induction; t0: baseline assessment before the first task block; t1: assessment after the first task block; t2: assessment after the second task block; WP: Word Pair test; WS: Word Scramble test.

The 2-way repeated measures ANOVA regarding participants’ change in negative affect showed significant main effects for the factors *test* (*F*_5.51,545.48_=5.13, *P*<.001, η_p_²=0.05) and *time* (*F*_1.18,116.62_=107.50, *P*<.001, η_p_²=0.52) as well as a significant *test×time* interaction effect (*F*_9.04,895.14_=7.96, *P*<.001, η_p_²=0.07). Tests of within-subject contrasts showed that negative affect scores significantly changed from t0 to t1 and from t0 to t2 in each stress-inducing test (MT, CN, AR, NS, WS, and WP) compared to the CC (control test) with medium to large effect sizes (Table 3). Post hoc pairwise comparisons for each test separately (Table 4) showed that negative affect scores significantly increased from t0 to t1 and from t0 to t2 in all tests except in the CC (control test), where there was only a significant increase from t0 to t2. There were significant increases in negative affect from t1 to t2 in the MT, NS, WS, WP, and CC, but not in the CN and AR. Figure 4 shows changes in negative affect. When controlling for *sample* (local university and crowdsourcing subsample; Table S1 in Multimedia Appendix 7), *gender* (female and male; Table S2 in Multimedia Appendix 7), and *employment* (employed and unemployed; Table S3 in Multimedia Appendix 7), all 3 showed no significant main or interaction effects on changes in negative affect scores by the SMSI tests. Thus, even controlling for *sample*, *gender*, or *employment*, our results showed that all stress-inducing tests of the SMSI robustly induced stress by eliciting negative affect, confirming our preregistered hypothesis.

**Table 3:** Contrast statistics of the 2-way repeated measures ANOVA for negative affect measured by the Positive and Negative Affect Schedule of the stress-inducing tests against the control test of the Salzburg Mobile Stress Induction from t0^a^ to t1^b^ and t2^c^ (N=100).

Negative affect		CC (CT)^d^ versus					
		MT^e^	CN^f^	AR^g^	NS^h^	WS^i^	WP^j^
**t0 versus t1**							
	*F* test (*df*)	29.02 (1, 99)	15.36 (1, 99)	29.07 (1, 99)	20.98 (1, 99)	32.25 (1, 99)	15.31 (1, 99)
	*P* value	<.001^k^	<.001^k^	<.001^k^	<.001^k^	<.001^k^	<.001^k^
	η_p_²^l^	0.23	0.13	0.23	0.18	0.25	0.13
**t0 versus t2**							
	*F* test (*df*)	42.06 (1, 99)	10.63 (1, 99)	26.23 (1, 99)	34.83 (1, 99)	33.67 (1, 99)	16.94 (1, 99)
	*P* value	<.001^k^	.002^k^	<.001^k^	<.001^k^	<.001^k^	<.001^k^
	η_p_²	0.30	0.10	0.21	0.26	0.25	0.15

^a^t0: baseline assessment before the first task block.

^b^t1: assessment after the first task block.

^c^t2: assessment after the second task block.

^d^CC (CT): Caesar Cipher test (control test).

^e^MT: Matrices test.

^f^CN: Cube Net test.

^g^AR: Arithmetic test.

^h^NS: Number Series test.

^i^WS: Word Scramble test.

^j^WP: Word Pair test.

^k^This footnote emphasizes significance.

^l^η_p_²=partial eta squared.

**Table 4:** Mean and SD of negative affect measured by the Positive and Negative Affect Schedule and Bonferroni-adjusted *P* values for post hoc pairwise comparisons between t0^a^, t1^b^, and t2^c^ for each test of the Salzburg Mobile Stress Induction separately and multivariate F statistics of time (t0, t1, and t2) within each test (N=100).

Negative affect			Tests												
			MT^d^		CN^e^		AR^f^		NS^g^		WS^h^		WP^i^		CC (CT)^j^
	t0, mean (SD)	11.12 (12.72)		16.38 (18.14)		15.20 (15.62)		11.04 (12.94)		10.66 (12.27)		12.35 (13.64)		15.30 (15.07)	
	t1, mean (SD)	21.01 (18.35)		22.76 (20.75)		25.49 (20.39)		19.93 (18.21)		19.91 (17.48)		19.59 (17.10)		16.54 (15.67)	
	t2, mean (SD)	25.02 (19.80)		24.08 (21.04)		27.28 (21.27)		25.59 (19.23)		22.58 (20.41)		22.50 (18.56)		18.52 (16.74)	
***P*** **value**															
	t0 versus t1	<.001^k^		<.001^k^		<.001^k^		<.001^k^		<.001^k^		<.001^k^		.63	
	t1 versus t2	<.001^k^		.27		.17		<.001^k^		.006^k^		.004^k^		.04^k^	
	t0 versus t2	<.001^k^		<.001^k^		<.001^k^		<.001^k^		<.001^k^		<.001^k^		.006^k^	
**Time**															
	*F* test (*df*)	45.94 (2, 98)		17.19 (2, 98)		33.07 (2, 98)		43.54 (2, 98)		33.69 (2, 98)		20.34 (2, 98)		5.82 (2, 98)	
	*P* value	<.001^k^		<.001^k^		<.001^k^		<.001^k^		<.001^k^		<.001^k^		.004^k^	
	η_p_²^l^	0.48		0.26		0.40		0.47		0.41		0.29		0.11	

^a^t0: baseline assessment before the first task block.

^b^t1: assessment after the first task block.

^c^t2: assessment after the second task block.

^d^MT: Matrices test.

^e^CN: Cube Net test.

^f^AR: Arithmetic test.

^g^NS: Number Series test.

^h^WS: Word Scramble test.

^i^WP: Word Pair test.

^j^CC (CT): Caesar Cipher test (control test).

^k^This footnote emphasizes significance.

^l^η_p_²=partial eta squared.

### Positive Affect

**Figure 5:**
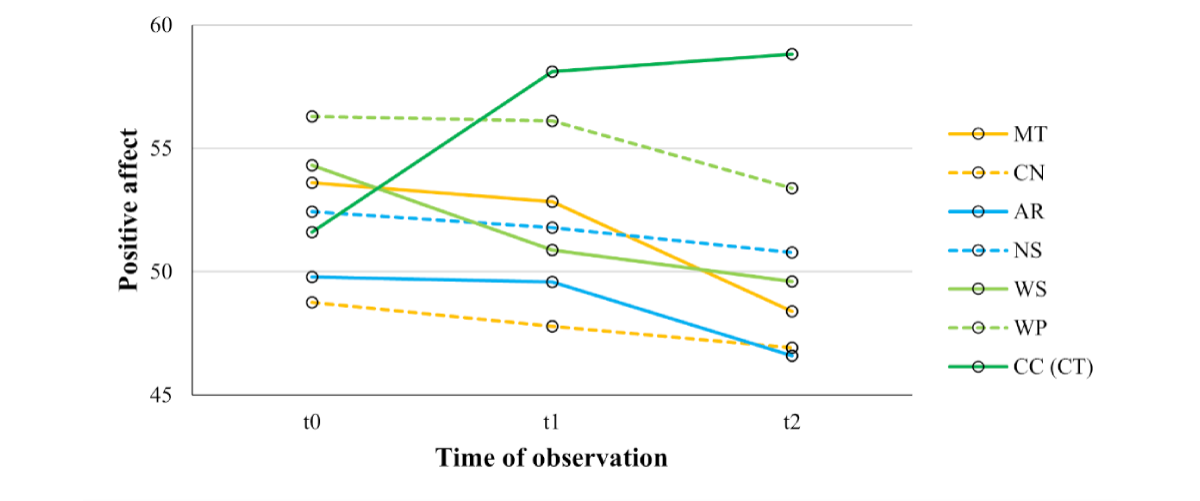
Positive affect was measured by the PANAS for each test of the SMSI across all participants (N=100) at t0, t1, and t2. Orange lines represent the figural tests (MT and CN), turquoise lines represent numerical tests (AR and NS), light green lines represent word-based tests (WS and WP), and the dark green line represents the word-based control test (CC (CT)). Positive affect scores significantly increased from t0 to t1 and from t0 to t2 in the CC (CT). Additionally, it significantly decreased from t0 to t1 in the WS, from t0 to t2 in the MT and NS (trend in the AR), as well as from t1 to t2 in the MT, AR, and WP. No significant changes occurred in positive affect from t0 to t1 in the MT, CN, AR, NS, and WP, from t0 to t2 in the CN, NS, and WP, and from t1 to t2 in the CN, NS, WS, and CC (CT). AR: Arithmetic test; CC (CT): Caesar Cipher test (control test); CN: Cube Net test; MT: Matrices test; NS: Number Series test; PANAS: Positive and Negative Affect Schedule; SMSI: Salzburg Mobile Stress Induction; t0: baseline assessment before the first task block; t1: assessment after the first task block; t2: assessment after the second task block; WP: Word Pair test; WS: Word Scramble test.

When examining the effects of the SMSI tests on positive affect, we found significant main effects of *test* (*F*_5.20,515.04_=5.76, *P*<.001, η_p_²=0.06) and *time* (*F*_1.26,124.74_=4.82, *P*=.02, η_p_²=0.05) and a significant *test×time* interaction effect (*F*_8.40,831.45_=8.08, *P*<.001, η_p_²=0.08). Tests of within-subject contrasts revealed significant changes in positive affect over time in all stress-inducing tests (MT, CN, AR, NS, WS, and WP) when compared to the CC (control test) with medium to large effect sizes (Table 5). Post hoc pairwise comparisons conducted for each test separately (Table 6) showed that positive affect scores significantly increased in the CC (control test) and significantly decreased in the WS from t0 to t1. Positive affect scores significantly increased in the CC (control test) and significantly decreased in the MT and WS (trend in the AR) from t0 to t2, and significantly decreased in the MT, AR, and WP from t1 to t2. Figure 5 illustrates changes in positive affect. When controlling for *sample* (local university and crowdsourcing subsample; Table S1 in Multimedia Appendix 8), we found a significant main effect of *sample* but no significant interactions with *test* or *time*, concluding in no deviation of the underlying effects. Participants in the crowdsourcing subsample reported overall higher positive affect compared to participants in the local university subsample (see Table S2 in Multimedia Appendix 8). When controlling for *gender* (female and male; Table S3 in Multimedia Appendix 8), we found no significant main or interaction effect of gender on changes in positive affect scores by the SMSI tests. When controlling for *employment* status, a significant main effect was found, but no significant interactions with *test* or *time*, and no deviation of the underlying effects were found (Table S4 in Multimedia Appendix 8). Employed participants reported overall higher positive affect compared to unemployed participants (Table S5 in Multimedia Appendix 8). While the SMSI stress-inducing tests partially decreased participants’ positive affect, participants reported increasing positive affect when conducting the CC (control test), when controlled for *sample*, *gender*, or *employment*.

**Table 5:** Contrast statistics of the 2-way repeated measures ANOVA for positive affect measured by the Positive and Negative Affect Schedule of the stress-inducing tests against the control test of the Salzburg Mobile Stress Induction from t0^a^ to t1^b^ and t2^c^ (N=100).

Positive affect		CC (CT)^d^ versus					
		MT^e^	CN^f^	AR^g^	NS^h^	WS^i^	WP^j^
**t0 versus t1**							
	*F* test (1, 99)	20.22	19.37	16.05	15.50	38.08	18.09
	*P* value	<.001^k^	<.001^k^	<.001^k^	<.001^k^	<.001^k^	<.001^k^
	η_p_²^l^	0.17	0.16	0.14	0.14	0.28	0.15
**t0 versus t2**							
	*F* test (1, 99)	41.57	23.05	35.74	19.06	48.12	25.67
	*P* value	<.001^k^	<.001^k^	<.001^k^	<.001^k^	<.001^k^	<.001^k^
	η_p_²	0.30	0.19	0.27	0.16	0.33	0.21

^a^t0: baseline assessment before the first task block.

^b^t1: assessment after the first task block.

^c^t2: assessment after the second task block.

^d^CC (CT): Caesar Cipher test (control test).

^e^MT: Matrices test.

^f^CN: Cube Net test.

^g^AR: Arithmetic test.

^h^NS: Number Series test.

^i^WS: Word Scramble test.

^j^WP: Word Pair test.

^k^This footnote emphasizes significance.

^l^η_p_²=partial eta squared.

**Table 6:** Mean and SD of positive affect measured by the Positive and Negative Affect Schedule and Bonferroni adjusted *P* values and effect sizes (ηp²) for post hoc pairwise comparisons between t0^a^, t1^b^, and t2^c^ for each test of the Salzburg Mobile Stress Induction separately and multivariate F statistics of time (t0, t1, and t2) within each test (N=100).

Positive affect			Tests												
			MT^d^		CN^e^		AR^f^		NS^g^		WS^h^		WP^i^		CC (CT)^j^
	t0, mean (SD)	53.60 (23.97)		48.76 (22.10)		49.79 (23.25)		52.43 (22.79)		54.32 (24.82)		56.29 (21.61)		51.61 (20.48)	
	t1, mean (SD)	52.83 (23.82)		47.79 (22.08)		49.59 (23.72)		51.78 (23.55)		50.88 (53.58)		56.11 (20.10)		58.11 (20.37)	
	t2, mean (SD)	48.40 (23.40)		46.91 (22.66)		46.59 (23.26)		50.78 (24.32)		49.60 (24.23)		53.38 (22.01)		58.81 (20.86)	
***P*** **value**															
	t0 versus t1	>.99		>.99		>.99		>.99		.004^k^		>.99		<.001^k^	
	t1 versus t2	<.001^k^		.87		.003^k^		.75		.41		.03^k^		>.99	
	t0 versus t2	.002^k^		.70		.099		.71		.001^k^		.24		<.001^k^	
**Time**															
	*F* test (*df*)	8.29 (2, 98)		0.77 (2, 98)		5.90 (2, 98)		0.97 (2, 98)		6.88 (2, 98)		3.67 (2, 98)		14.11 (2, 98)	
	*P* value	<.001^k^		.47		.004^k^		.38		.002^k^		.04^k^		<.001^k^	
	η_p_²^l^	0.15		0.02		0.11		0.02		0.12		0.06		0.22	

^a^t0: baseline assessment before the first task block.

^b^t1: assessment after the first task block.

^c^t2: assessment after the second task block.

^d^MT: Matrices test.

^e^CN: Cube Net test.

^f^AR: Arithmetic test.

^g^NS: Number Series test.

^h^WS: Word Scramble test.

^i^WP: Word Pair test.

^j^CC (CT): Caesar Cipher test (control test).

^k^This footnote emphasizes significance.

^l^η_p_²: partial eta squared.

## Discussion

### Principal Findings

This research aimed to develop and validate new paradigms for stress inductions. The introduction of the SMSI provides a new set of tools for repeatedly inducing stress in everyday life. In this preregistered study, we evaluated the potential effectiveness and feasibility of the SMSI to remotely induce stress in an ambulatory, digital framework using a smartphone app. Starting from the initial survey and installation, a large sample of participants performed the 7 SMSI tests over 4 days in their daily lives, without requiring a laboratory setting or participant-investigator interactions. We compared the effectiveness of the 6 stress-inducing tests (MT, CN, AR, NS, WS, and WP) with the similarly designed control test (CC). Participants reported an increase in negative affect in all stress-inducing tests of the SMSI compared to the control test. However, participants’ positive affect decreased in the MT, AR, WS, and WP, while the CC control test induced an increase in positive affect.

We found support for our hypothesis that participants experienced significantly more negative affect after completing the stress-inducing tests compared to the control test, with medium (CN) to large (MT, AR, NS, WS, and WP) effect sizes. Participants reported the strongest increase in negative affect after the first task block of the stress-inducing tests, which continued to increase significantly in the MT, NS, WS, and WP. Although the rise in negative affect in the CN and AR leveled off after the first task block, participants’ negative affect remained significantly higher after the second task block compared to the baseline. Although the CC slightly increased negative affect over time, this increase occurred later in the procedure and may be attributed to exposure to an ongoing participation in a performance test. Overall, the effect of the CC on negative affect was minimal compared to the stress-inducing tests. Among these, the MT exhibited the highest stress-inducing potential, followed by the NS, WS, AR, WP, and CN. Although the crowdsourcing sample received a different primary framing for the entry survey, no sample effect and no change in the overall effects of the SMSI on negative affect were found. This indicates that participants’ perception of the smartphone study’s objective did not differ between the 2 subsamples. Similarly, no significant differences in negative affect were found in being female and male or in employed and unemployed participants.

Overall, the SMSI provides a feasible way to induce acute stress responses outside the laboratory, which is simple to implement in various smartphone apps (eg, m-Path [17]). These findings outline the potential of cognitive tasks as stress induction paradigms without components of social-evaluative threat, such as the TSST [8] or the DST [13] applied. This aligns with the latter’s findings that social comparison characteristics contributed less to the stress-inducing process in the DST study. The incremental value of the SMSI lies in its potential for repetitive usage, as it offers 6 different stress paradigms with comparable effectiveness to its mobile stress-inducing predecessor, the DST. To accurately observe stress occurrences, ambulatory stress research often requires high measurement frequencies, thereby exerting high demands on both participants and researchers [6]. The SMSI reduces risks of habituation effects [12] and enables researchers to tailor various stress inductions to their specific needs in their ambulatory studies (eg, ecological momentary assessments [4] or ecological momentary interventions [5]) by freely choosing from the provided list of tests of the toolbox.

Challenging and demanding tasks—whether encountered in daily life, or in our case, through participation in the SMSI tests—not only increase negative stress but also affect positive experiences as well [28]. Our results showed that participants’ positive affect changed significantly during the stress-inducing tests compared to the neutral control test. In 4 of the 6 stress-inducing tests (MT, AR, WS, and WP), positive affect significantly decreased during the procedure. When controlling for sampling procedure and employment status, we found significant main effects, although no significant interaction effects emerged between the SMSI tests and measurement times. Nevertheless, the main findings remained stable. Overall, participants from the crowdsourcing subsample reported higher positive affect scores than the local university subsample, likely due to differences in compensation. While the local university participants received participation credits, crowdsourcing participants were paid, which may have enhanced their overall experience of this study. Similarly, employed participants reported overall higher positive affect than unemployed participants, which might be attributed to habituation of completing tasks beyond the university (ie, in the workplace). Similar to these results on negative affect, no significant effect for gender differences was found.

While it has been theorized that demanding tasks can enhance positive outcomes (eg, positive emotions) [28], one momentary assessment study revealed stressors—such as heightened job, private life, or study-related demands—were related to lower positive stress responses [29]. Contrary to the stress-inducing SMSI tests, participants reported an increase in positive affect after conducting the CC (control test), similar to the DST findings [13]. Although the CC was established as a control test for negative affect and negative stress, these results might indicate that the CC could represent a new type of stress paradigm: a positive, eustress-inducing paradigm. Lazarus transactional model of stress [30] was among the first theoretical approaches in psychology that distinguished stress into its 2 types of distress and eustress. In particular, eustress occurs when a stressor is appraised not as a threat or hindrance but as a challenge—thereby inducing a feeling of accomplishment and healthy consequences. Building upon the transactional model of stress, the holistic stress model [31] proposed that stress responses are dynamic and can be of a physiological, behavioral, or psychological nature. This model highlights that emotions—such as positive affect—are among the preferred indicators of eustress, as they reflect feelings of activeness, enthusiasm, and alertness. The experience of eustress following the CC may be due to participants perceiving the task as challenging yet solvable, as it was intentionally designed. These results open new possibilities for positive psychology research by enabling the direct investigation of eustress responses and moderating traits (eg, skills and resources) or eustress-enhancing strategies (eg, savoring) [31]. The psychological literature contains far fewer positive mood induction procedures than negative ones [32], and to our knowledge, this is the first method using cognitive tasks with the potential to elevate not only positive affect but also eustress by applying challenging yet solvable tasks. However, this assumption still requires further investigation. Although positive affect can serve as an indicator for eustress [31], the current findings allow us to only hypothesize the eustress-inducing potential of the CC (and potential adapted tests). Follow-up studies should deviate from traditional affect-based stress assessments and use dedicated distress and eustress measures to holistically capture changes in psychological stress. A holistic assessment of stress is highly recommended as distress and eustress are not mutually exclusive and might be simultaneously experienced [31]. The recently published Di-Eu-Stress State Scale [29] presents a concise and thoroughly validated questionnaire of both negative and positive stress states, which is specifically well-suited for ambulatory research. Future research might benefit threefold from combining the SMSI and the Di-Eu-Stress State Scale: (1) gather evidence of an actual eustress paradigm; (2) establish further validation of the distress-inducing potential of the SMSI beyond assessments of negative affect; and (3) gain detailed insights about positive and negative stress experiences and how the individual’s perception varies in everyday life.

### Feasibility of the SMSI in Ambulatory Research

The main objective of the SMSI is to provide a feasible stress induction paradigm that can be repeatedly implemented in ambulatory studies. Therefore, its feasibility in such research is essential. This study evaluated the SMSI’s applicability within its intended ambulatory framework.

Similar to many other ambulatory research projects, compliance with study prompts posed certain challenges in this study. Many participants who initially registered for this study (129/229, 56.3%) missed at least 1 mobile prompt to participate in a scheduled test. We sought possible indicators for missing a test in our data. However, neither sociodemographic nor personality characteristics nor global perceived stress explained the differences between completionists and noncompletionists. Repeated measurements and study interactions are prone to increasing participant burden due to their time-consuming nature and their inherent interference with participants’ daily lives [33]. Consequently, several meta-analyses [34-38] have investigated the characteristics of compliance in ambulatory assessment. In 3 of these meta-analyses [35,37,38], participants’ compliance was positively related to age, which could explain some of the compliance rate in this study, as the sample consisted mainly of younger adults. However, other findings reported no such association [34,36]. Stress inductions are inherently intensive, a factor researchers should consider when applying them in ambulatory studies. According to Ottenstein and Werner [34], compliance rates are lower in intervention studies than in studies without interventions. Additionally, a recent study evaluating the effects of intensity in ambulatory assessment studies showed that the questionnaire length (30- vs 60-item questionnaires) was positively related to participant burden and negatively related to compliance [39]. Although compliance is considerably challenging in ambulatory research, there are methods to increase consecutive participation. Pronounced reminders (eg, alarms or numerous prompts) can effectively increase compliance [38], as participants’ primary reason for not responding to prompts is seldom a lack of motivation but rather their inability to reply at the designated time [39]. In this study, we used only 1 reminder, which was sent 15 minutes after the prompt. In future studies, we plan to increase the number of reminders to further increase participation likelihood. Taken together, the attrition rate in this study reflects typical participant behavior in ambulatory research and is unlikely to indicate systematic biases due to the SMSI. Therefore—as with most ambulatory studies—future SMSI applications in ambulatory studies should carefully plan their study procedures per literature recommendations to enhance participant compliance (eg, shorter length of study period or monetization as incentives) and assess participation motivation to gain further insights into this concept [34].

Although participants had the option to cancel a test procedure at any time by closing the app, all participants who initiated a test completed it in full. In contrast, the DST study [13] had a shorter duration than the SMSI tests, but over half of the initial participants dropped out during the test procedure. Even a recent meta-analysis [34] showed that the percentage of dropouts was higher in ambulatory studies with interventions than in studies without interventions. Although the compliance rate during this study’s procedure presented similar challenges to those in traditional ambulatory assessment studies, the 0% dropout rate during test administration emphasizes the SMSI’s practical utility in ensuring psychological stress responses in ambulatory study designs.

### Limitations and Future Research

This study represents the first evaluation of the SMSI’s effectiveness outside the laboratory, using behavioral data to examine changes in stress perception. However, while it is known that acute stress affects the entire central nervous system either directly or indirectly, certain areas of the brain play crucial roles in the stress system. Therefore, future validation studies should rely on questionnaire data while examining major stress systems, such as the hypothalamic-pituitary-adrenal axis or the more rapidly responsive autonomic nervous system [40]. For example, assessing neuroendocrine stress markers (eg, salivary cortisol and α-amylase [41]) and cardiovascular processes (eg, heart rate and heart rate variability [42]), respectively, provides noninvasive methods to objectively investigate the stress response. Although some studies indicated that speech tasks without social evaluative threats do not increase salivary cortisol [43], previous ambulatory assessment [44] has shown that heart rate and heart rate variability respond to stressful cognitive tests in both individuals with anxiety disorders and in healthy individuals. Consequently, future validation studies must use different methods of physiological measures to better understand the SMSI’s impact on the human organism. Using ambulatory saliva sampling [45] or portable electrocardiogram sensors [46,47] could enable physiological stress assessments of salivary cortisol and heart rate outside laboratory settings. This approach would reduce participant burden and enable examination of stress reactivity mechanisms in participants’ daily lives [6].

Previous stress induction paradigms that use cognitive stimuli, such as the Montreal Imaging Stress Task [14] in laboratory settings or the arithmetic calculation task of the DST [13] in ambulatory applications, present participants with their performance rates alongside a display of the average (mostly artificially preset) performance rate of potential peers. This approach aims to enhance the effectiveness of stress induction through social evaluative threat. The SMSI operates solely on cognitive stressors, which simplifies programming and implementation in various software. Although a prospective extension of the SMSI, including social comparisons, could provide an interesting avenue to explore the respective effects and interplay between cognitive stress stimuli and social evaluative threats. Both approaches—cognitive stressors with or without social components—can be applied to different areas of everyday life. In particular, cognitive demands have increased due to digitalization in both work and daily life [48]. The boundary between work and personal life became blurred, leading to evolving demands on digital work and a rising need for corresponding digital skills [49]. These new cognitive demands present new challenges in daily lives, and depending on the specific cognitive demand, they can have either negative or positive consequences [48]. Thus, the SMSI can be used to evoke similar stress responses and investigate individuals’ reactions to either stress type outside the laboratory. De Calheiros Velozo et al [16] demonstrated that recovery from stress induced in the laboratory was linked to stress reactivity in daily life. However, in their study, stressful events were scarce and difficult to detect. By presenting targeted stressors, the SMSI addresses this gap and enables the standardized investigation of individuals’ stress response, reactivity, and recovery in everyday life.

The promising results found in this study are limited in their transferability to other populations, as the SMSI remains validated only on university students. While students are generally more educated than the general population, they are vulnerable to many demands that lead to stress. Similar to how digitalization has blurred work-life boundaries [49], students’ academic, social, and work lives are constantly blending, thereby potentially inducing a maladaptive work-life balance that contributes to heightened experiences of stress, anxiety, and depression symptoms [50,51]. To enhance SMSI’s validity, comparable studies comprising healthy employees should be conducted to provide additional insights into the SMSI’s effectiveness in other populations. As cognitive demands rise in the occupational field due to digitalization [48], SMSI procedures should be easily applicable to these individuals. For example, the effects observed in this study’s sample did not differ between employed and unemployed participants. However, the SMSI’s feasibility should be tested on individuals with lower levels of digital literacy and less experience with cognitive demands. Additionally, the SMSI may be useful for clinical samples. However, as cognitive function is often impacted by various symptomologies (eg, in hospitalized older patients [52], sleep-disordered breathing [53], first-episode of depression [54])—the SMSI’s nature as a cognitive performance test should be considered.

Despite these challenges, the SMSI potentially allows for conducting stress inductions in different nations, as 4 SMSI tests (MT, CN, AR, and NS) are inherently nonverbal. However, the 3 word-based tests (WS, WP, and the control test CC) depend on language comprehension skills and are currently only validated in German. As a first step toward broader applicability, we have developed an English version of these tests to offer the SMSI more globally and are currently validating the entire SMSI with a population of native English speakers (for the test materials of both languages, see the SMSI Test Material Availability section). The overall testing procedure mirrors the German study. To ensure comparable effects, the tasks of the word-based tests were reconstructed to align precisely with the original tasks, while considering the respective population and language comprehension abilities. Further adaptations in additional languages would also enable standardized investigations into cross-cultural differences of acute stress responses [32] in participants’ daily lives. The digital nature of the SMSI additionally alleviates investigations of larger sample sizes in a short time compared to strenuous laboratory experiments.

### Conclusions

This research aimed to develop and validate a toolbox of newly conceptualized stress paradigms for inducing stress responses in study participants’ daily lives, without requiring participant-investigator interactions. The SMSI’s advantage is its ability to repeatedly apply stress inductions, thereby enabling a standardized examination of consecutive stress reactions outside laboratory settings. This study provided the first promising results on the potential of cognitive tasks to induce psychological stress in participants’ natural environment using smartphone apps.

In addition to demonstrating the effectiveness of the 6 stress-inducing tests and the SMSI’s feasibility, the potential of the CC to elicit positive emotions offers new possibilities for stress research. By making the SMSI and its test materials open access (see SMSI Test Material Availability section), researchers can easily integrate and adapt mobile stress induction in their (ambulatory) studies as required. Future studies will further explore the SMSI’s potential to induce both negative distress and positive eustress in ambulatory research and the SMSI’s effect on physiological stress systems.

## Appendix

Multimedia Appendix 1 Example screenshots of the word-based tests of the Salzburg Mobile Stress Induction (SMSI) in the German language used in this study: Task of the Word Scramble test (Figure S1), word pair memory list of the Word Pair test (Figure S2), task of the Word Pair test (Figure S3), and task of the Caesar Cipher control test (Figure S4).

Multimedia Appendix 2 Video illustrating the complete procedure of the Arithmetic test of the Salzburg Mobile Stress Induction. The test was performed in German on a Google Pixel 6a (Android version 15) smartphone in the m-Path App, as used in this study. The video has been modified to 2x playback speed. For proper inspection or reading of specific parts, the video can be paused. Screen recordings were done by the first author in April 2025.

Multimedia Appendix 3 Step-by-step image-based instruction on how to download the m-Path app used in this study on the participants’ own smartphone devices as well as on how to register and connect to the research account within the m-Path app. Instructions are in the German language (an English version of the step-by-step image-based instruction used in the currently ongoing validation study of the SMSI English version Mobile Stress Induction is available from the authors).

Multimedia Appendix 4 Table S1: Table of Cronbach α of the Positive and Negative Affect Schedule subscales—negative affect (5 items) and positive affect (5 items)—for each test of the Salzburg Mobiles Stress Induction at baseline (t0), after the first task block (t1), and after the second task block assessments (t2; N=100).

Multimedia Appendix 5 Table S1: Table of ANOVA statistics of comparisons of participants at baseline (N=212) who dropped out of the study or missed a test (n=117) and participants who completed all 7 tests (n=95) of initial survey measures of sociodemographic and personality characteristics, and perceived stress. Participants who dropped out of the study or missed a test did not differ significantly from participants who completed all 7 Salzburg Mobile Stress Induction tests.

Multimedia Appendix 6 Table S1: Table of the sociodemographic characteristics of the total sample and split between the local university and crowdsourcing subsamples.

Multimedia Appendix 7 Control analyses tables of the 2-way repeated measures ANOVAs and contrast statistics for negative affect of the stress-inducing tests (Matrices test [MT], Cube Net test [CN], Arithmetic test [AR], Number Series test [NS], Word Scramble test [WS], and Word Pair test [WP]) against the control test (Caesar Cipher test [CC]) of the Salzburg Mobile Stress Induction (test) from baseline (t0) to after the first task block (t1) and after the second task block assessments (t2; time) controlling for sample (local university, n=69, and crowdsourcing subsample, n=31; N=100; Table S1), gender (60/96, women and 34/96, men; N=96; Table S2), and employment (employed, n=51, and unemployed, n=44; N=96; Table S3) as between-subject factor.

Multimedia Appendix 8 Control analyses tables of the 2-way repeated measures ANOVAs and contrast statistics for positive affect of the stress-inducing tests (Matrices test [MT], Cube Net test [CN], Arithmetic test [AR], Number Series test [NS], Word Scramble test [WS], and Word Pair test [WP]) against the control test (Caesar Cipher test [CC]) of the Salzburg Mobile Stress Induction (test) from baseline (t0) to after the first task block (t1) and after the second task block assessments (t2; time) controlling for sample (local university, n=69, and crowdsourcing subsample, n=31; N=100; Table S1; Table S2 shows Mean and SD scores separated for the total, local university, and crowdsourcing sample), gender (60/96, women and 34/96, men; N=96; Table S3), and employment (employed, n=51, and unemployed, n=44; N=96; Table S4; Table S5 shows Mean and SD scores separated for total, employed and unemployed samples) as between-subject factor.

## Abbreviations

AR: Arithmetic test
CC: Caesar Cipher test
CN: Cube Net test
DST: Digital Stress Test
MT: Matrices test
NS: Number Series test
PANAS: Positive and Negative Affect Schedule
SMSI: Salzburg Mobile Stress Induction
TSST: Trier Social Stress Test
WP: Word Pair test
WS: Word Scramble test

## Data Availability

The Salzburg Mobile Stress Induction (SMSI) test materials, datasets, and data processing scripts are openly available in the Open Science Framework depository [22].
