# Supplementary material for: Digital Stress Induction in Daily Life Using the Salzburg Mobile Stress Induction (SMSI): Development and Ambulatory Evaluation Study
Source: J Med Internet Res. 2025 Sep 18;27:e75785. doi: 10.2196/75785 (PMC12491893; doi:10.2196/75785)

## Multimedia Appendix 1

**Figure S1.** Example screenshot of the word-based Word Scramble test of the Salzburg Mobile Stress Induction (SMSI) in the German language used in this study.


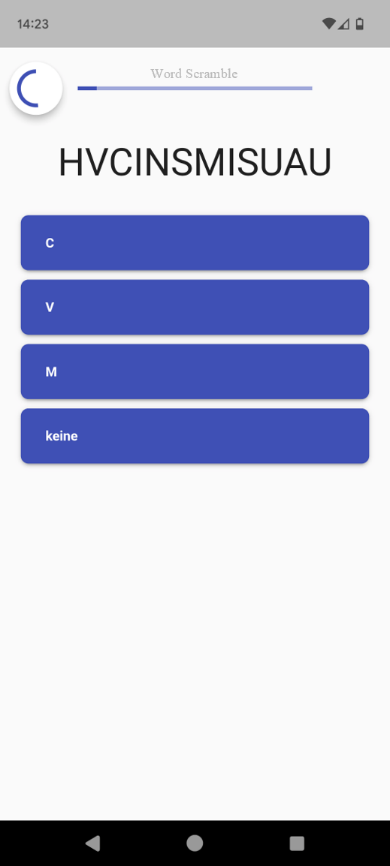


**Figure S2.** Example screenshot of the fourth word pair memory list of the word-based Word Pair test of the Salzburg Mobile Stress Induction (SMSI) in the German language used in this study.


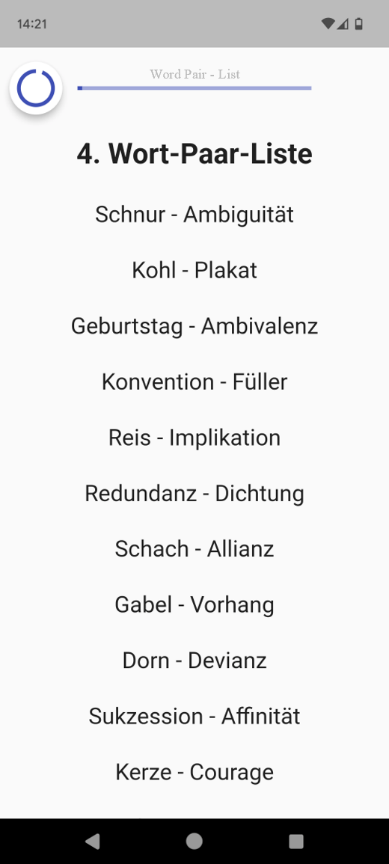


**Figure S3.** Example screenshot of a task of the word-based Word Pair test of the Salzburg Mobiles Stress Induction (SMSI) in the German language used in this study.


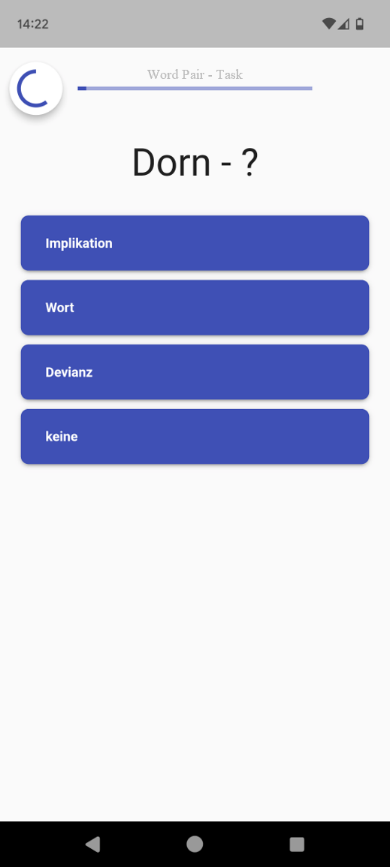


**Figure S4.** Example screenshot of a task of the word-based Caesar Cipher control test of the Salzburg Mobile Stress Induction (SMSI) in the German language used in this study.


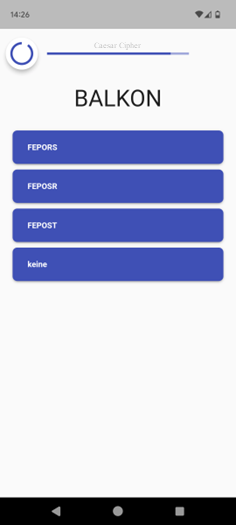

Supplement: Multimedia Appendix 1 [file jmir_v27i1e75785_app1.doc]
