# Supplementary material for: Digital Stress Induction in Daily Life Using the Salzburg Mobile Stress Induction (SMSI): Development and Ambulatory Evaluation Study
Source: J Med Internet Res. 2025 Sep 18;27:e75785. doi: 10.2196/75785 (PMC12491893; doi:10.2196/75785)

## Multimedia Appendix 3

**m-Path Installation am eigenen Smartphone**

Im nächsten Schritt folgt die Installation der für die weitere Studie benötigte m-Path-App auf Ihrem eigenen Smartphone.

Halten Sie hierfür bitte Ihr Smartphone bereit.

Um die verschiedenen Erhebungen der Eingangserhebung und Smartphone-Teile in Verbindung bringen zu können, benötigen wir einen individuellen **Versuchspersonen-Code**.
Dieser wird nach der Verknüpfung der verschiedenen Erhebungen aus den Datensätzen gelöscht und es wird daraus kein Rückschluss auf Ihre Person möglich sein.

**WICHTIG: Dieser Code wird auch benötigt, um Ihre Entlohnung zu erhalten. Geben Sie diesen Code auch in m-Path als Ihren Benutzernamen an (Anleitung für m-Path Installation siehe unten)!**

Bitte erstellen Sie zunächst Ihren persönlichen **Versuchspersonen-Code**. Dieser soll sich wie folgt zusammensetzen:

1. Die **ZWEI** ersten Buchstaben des Vornamens Ihrer Mutter in Großbuchstaben (Bsp.: **An**na --> **AN**)

2. Die **ZWEI** ersten Buchstaben des Vornamens Ihres Vaters in Großbuchstaben (Bsp.: **Be**rnd --> **BE**)

3. Ihr Geburtsmonat in **Zahlen** (Bsp.: September --> **09**)

Der Beispielcode in diesem Falle wäre dann: **ANBE09**

Bitte benutzen Sie dabei volle Namen und keine Spitznamen oder Abkürzungen (z.B. Anton statt Toni).

Als nächstes bitten wir Sie, die m-Path App auf Ihrem Smartphone zu installieren. Für die Installation benötigen Sie Ihren eben angegebenen Versuchspersonen-Code.

**Anleitung zur Installation der mPath App:**

**1) Laden Sie die m-Path App auf Ihr Smartphone.**

Die m-Path App ist kostenlos und kann unter folgenden Links gefunden werden:

[App Store](https://apps.apple.com/de/app/m-path/id1469274164) [Google Play](https://play.google.com/store/apps/details?id=io.m_Path.kuleuven&gl=BE&pli=1)


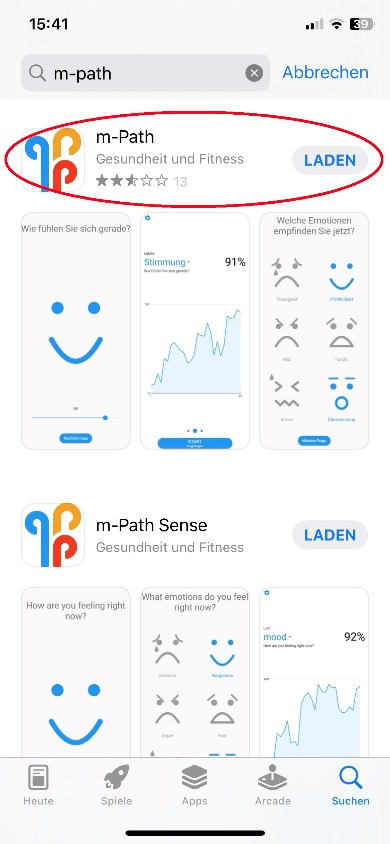

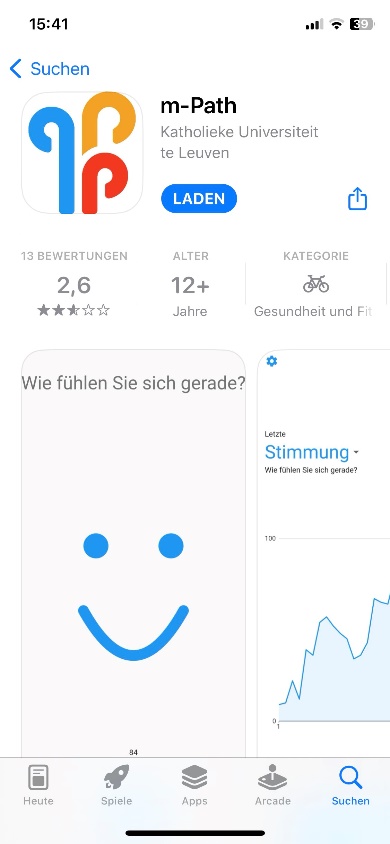


**2) Tragen Sie als „Alias“ Ihren Versuchspersonencode ein.**

Tragen Sie **NICHT** Alias ein.

Verwenden Sie unbedingt den selben 6-stelligen Versuchspersonencode in der m-Path App wie auch in der LimeSurvey-Umfrage!


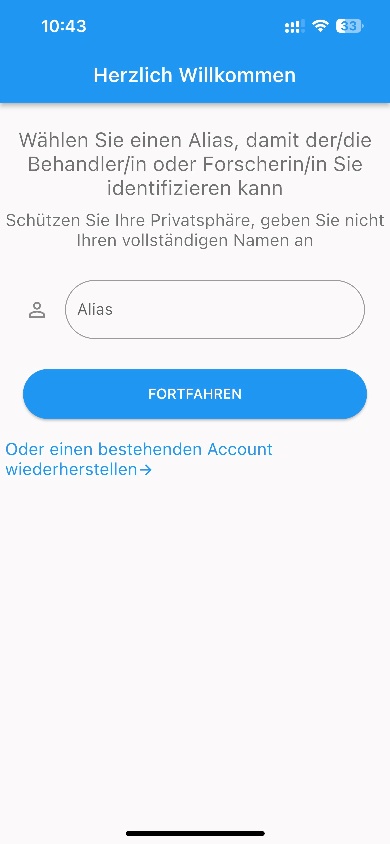


**ERINNERUNG:**

**Ihr Versuchspersonen-Code setzt sich wie folgt zusammensetzen:**

1. Die **ZWEI**ersten Buchstaben des Vornamens Ihrer Mutter in Großbuchstaben (Bsp.: **An**na --> **AN**)

2. Die **ZWEI**ersten Buchstaben des Vornamens Ihres Vaters in Großbuchstaben (Bsp.: **Be**rnd --> **BE**)

3. Ihr Geburtsmonat in **Zahlen**(Bsp.: September -->**09**)

Der Beispielcode in diesem Falle wäre dann: **ANBE09**

**3) Lesen und akzeptieren Sie die Bedingungen.**


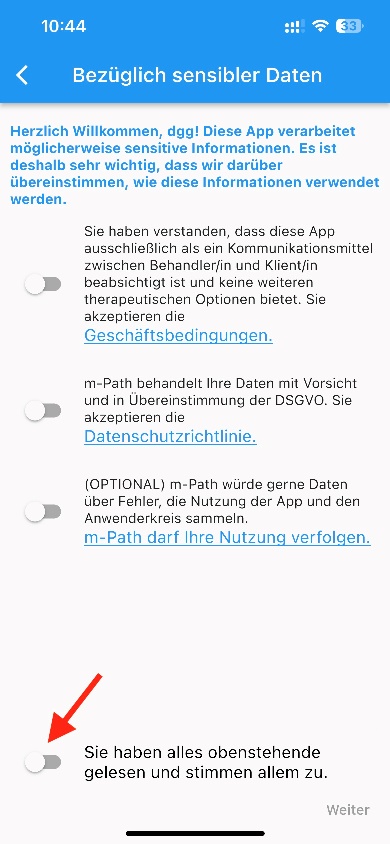


**4) Speichern Sie sich Ihren Wiederherstellungscode ab:**
Der Wiederherstellungscode ist ein Code, der Ihren Account auf einem neuen Gerat oder nach Reinstallation der App wiederherstellen kann. **Speichern Sie sich deshalb diesen ab** *(z.B. in einer Notiz oder als Screenshot auf Ihrem Smartphone)***.**


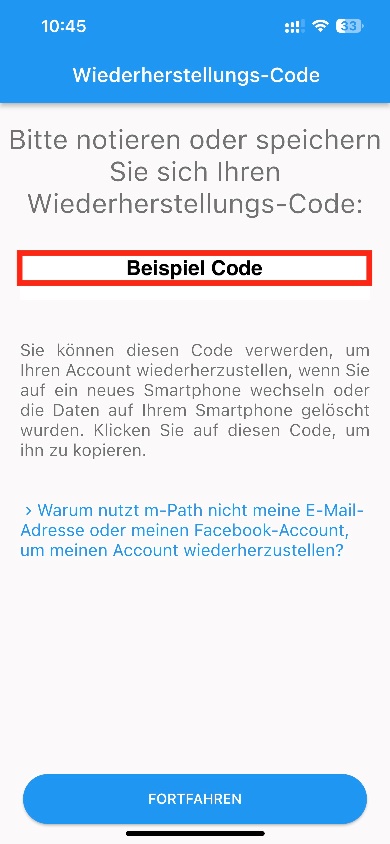


**5) Fügen Sie unsere Forschungsarbeit hinzu, indem Sie folgenden Code eingeben:**

**AcC-cOdE^*^**


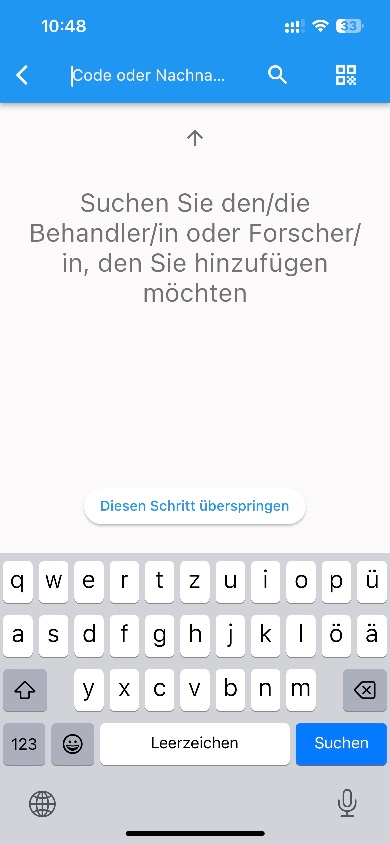

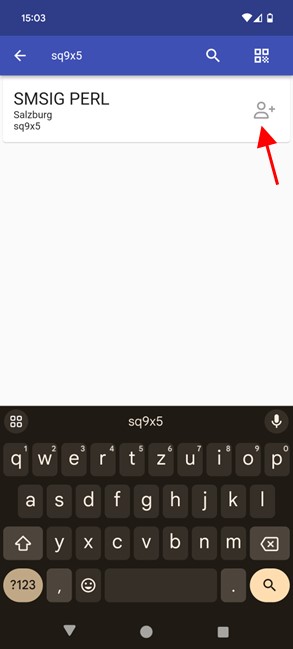

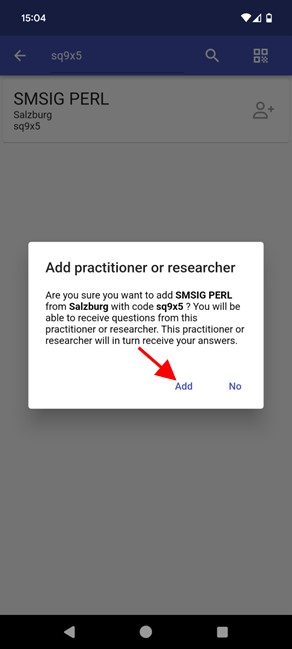


**code and screenshots must be adjusted to your respective m-Path researcher account*

**6) Wählen Sie „Testen“ aus.**


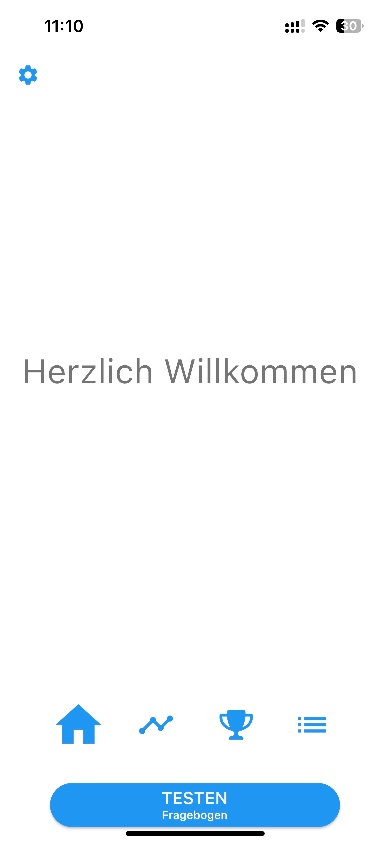


**Bei weiteren Fragen wenden Sie sich bitte an:** [**SMSIG.PERL.PLUS@gmail.com**](mailto:smsig.perl.plus@gmail.com)

**Haben Sie m-Path auf Ihrem Smartphone installiert, Ihren Versuchspersonencode als Benutzernamen und uns als Forscher hinzugefügt?**


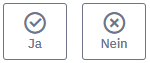

Supplement: Multimedia Appendix 3 [file jmir_v27i1e75785_app3.doc]
