# Supplementary material for: Digital Stress Induction in Daily Life Using the Salzburg Mobile Stress Induction (SMSI): Development and Ambulatory Evaluation Study
Source: J Med Internet Res. 2025 Sep 18;27:e75785. doi: 10.2196/75785 (PMC12491893; doi:10.2196/75785)
Supplement: Multimedia Appendix 4 [file jmir_v27i1e75785_app4.doc]

## Multimedia Appendix 4

**Table S1.** Table of Cronbach α of the Positive and Negative Affect Schedule (PANAS) subscales ***negative affect*** (5 items) and ***positive affect*** (5 items) for each test of the Salzburg Mobiles Stress Induction and at baseline (t0), after the first task block (t1) and after the second task block assessments (t2; N=100).

| **Negative affect - PANAS** | | | |
| --- | --- | --- | --- |
| Test | Cronbach α | | |
|  | t0 | t1 | t2 |
| Matrices test | .80 | .82 | .79 |
| Cube Net test | .86 | .87 | .85 |
| Arithmetic test | .81 | .82 | .82 |
| Number Series test | .77 | .84 | .81 |
| Word Scramble test | .80 | .84 | .88 |
| Word Pair test | .81 | .81 | .81 |
| Caesar Cipher control test | .77 | .80 | .81 |
| **Positive affect - PANAS** | | | |
| Test | Cronbach α | | |
|  | t0 | t1 | t2 |
| Matrices test | .91 | .91 | .91 |
| Cube Net test | .87 | .89 | .90 |
| Arithmetic test | .90 | .91 | .90 |
| Number Series test | .91 | .91 | .93 |
| Word Scramble test | .92 | .93 | .93 |
| Word Pair test | .87 | .87 | .88 |
| Caesar Cipher control test | .88 | .88 | .89 |
