# Supplementary material for: Digital Stress Induction in Daily Life Using the Salzburg Mobile Stress Induction (SMSI): Development and Ambulatory Evaluation Study
Source: J Med Internet Res. 2025 Sep 18;27:e75785. doi: 10.2196/75785 (PMC12491893; doi:10.2196/75785)
Supplement: Multimedia Appendix 5 [file jmir_v27i1e75785_app5.doc]

## Multimedia Appendix 5

**Table S1.** Table of ANOVA statistics of comparisons of participants at baseline (N=212) who dropped out of the study or missed a test (n=117) and participants who completed all seven tests (n=95) of initial survey measures of socio-demographic and personality characteristics, and perceived stress. Participants who dropped out of the study or missed a test did not differ significantly from participants who completed all seven SMSI tests. η²=eta squared; BFI=Big Five Inventory; BTPS=Big Three Perfectionism Scale; PSQ=Perceived Stress Questionnaire 20.

|  |  |  | *F* test (1, 210) | *P* value | η² |
| --- | --- | --- | --- | --- | --- |
| **Socio-demographic data** | | | | | |
|  | Gender | | 1.23 | .27 | 0.01 |
|  | Age | | 0.10 | .75 | 0.00 |
|  | Field of study | | 1.07 | .30 | 0.01 |
|  | Nationality | | 0.02 | .88 | 0.00 |
|  | Highest education | | 0.82 | .37 | 0.00 |
|  | Academic degree sought | | 0.81 | .37 | 0.00 |
|  | Type of university | | 1.23 | .27 | 0.01 |
|  | Country of study/university | | 0.31 | .58 | 0.00 |
|  | Study extent (full vs. part time) | | 1.43 | .23 | 0.01 |
|  | Semester of study (sum of semesters) | | 0.78 | .38 | 0.00 |
|  | Planned ECTS credits for the current semester | | 0.22 | .64 | 0.00 |
|  | Study type (attendance vs. online vs. both) | | 1.55 | .22 | 0.01 |
|  | Employed besides studying (detailed) | | 0.33 | .56 | 0.00 |
|  | Employed besides studying (No or Yes) | | 0.12 | .73 | 0.00 |
|  | Previous professional qualification or study | | 1.10 | .30 | 0.01 |
| **Personality characteristics** | | | | | |
|  | *Big five personality traits* | | | | |
|  |  | BFI Conscientiousness Scale | 3.08 | .08 | 0.01 |
|  |  | BFI Extraversion Scale | 2.17 | .14 | 0.01 |
|  |  | BFI Agreeableness Scale | 0.30 | .59 | 0.03 |
|  |  | BFI Openness Scale | 0.01 | .94 | 0.00 |
|  |  | BFI Neuroticism Scale | 0.22 | .64 | 0.00 |
|  | *Self-Efficacy* | | | | |
|  |  | General Self-Efficacy Scale | 0.00 | .99 | 0.00 |
|  | *Perfectionism* | | | | |
|  |  | BTPS Rigid Perfectionism | 2.84 | .09 | 0.01 |
|  |  | BTPS Self-Critical Perfectionism | 1.17 | .28 | 0.01 |
|  |  | BTPS Narcissistic Perfectionism | 3.37 | .07 | 0.02 |
| **Perceived Stress** | | | | | |
|  | Global Stress Score - PSQ | | 0.22 | .64 | 0.00 |
|  | Demands subscale - PSQ | | 0.22 | .64 | 0.00 |
|  | Worries subscale - PSQ | | 0.22 | .64 | 0.00 |
|  | Joy subscale - PSQ | | 0.22 | .64 | 0.00 |
|  | Tension subscale - PSQ | | 0.22 | .64 | 0.00 |
