# Supplementary material for: Digital Stress Induction in Daily Life Using the Salzburg Mobile Stress Induction (SMSI): Development and Ambulatory Evaluation Study
Source: J Med Internet Res. 2025 Sep 18;27:e75785. doi: 10.2196/75785 (PMC12491893; doi:10.2196/75785)
Supplement: Multimedia Appendix 6 [file jmir_v27i1e75785_app6.doc]

## Multimedia Appendix 6

**Table S1.** Table of the socio-demographic characteristics of the total sample and split between the local university and crowdsourcing subsamples.

| Characteristics | | Total sample (N=100)^a^ | Local university subsample (n*=*69) | Crowdsourcing subsample (n=31) |
| --- | --- | --- | --- | --- |
| **Missing demographic data, n (%)^b^** | | 5 | 3 (4) | 2 (7) |
| **Gender, n (%)** | |  |  |  |
|  | Male | 34 | 15 (22) | 19 (61) |
|  | Female | 60 | 50 (73) | 10 (32) |
|  | Non-binary | 1 | 1 (1) | 0 (0) |
| **Age (years), mean (SD)** | | 24.43 (6.21) | 22.14 (4.49) | 29.66 (6.49) |
| **Nationality, n (%)** | |  |  |  |
|  | Germany | 64 | 40 (58) | 24 (77) |
|  | Austria | 23 | 20 (29) | 3 (10) |
|  | Swiss | 2 | 1 (1) | 1 (3) |
|  | Other | 1 | 5 (7) | 1 (3) |
| **Education, n (%)** | |  |  |  |
|  | High school | 61 | 49 (71) | 12 (39) |
|  | Bachelor | 25 | 13 (19) | 12 (30) |
|  | Master | 6 | 3 (4) | 3 (10) |
|  | Other | 3 | 1 (1) | 2 (7) |
| **Field of study, n (%)** | |  |  |  |
|  | Natural sciences | 55 | 53 (77) | 2 (7) |
|  | Languages, cultural and human sciences | 11 | 5 (7) | 6 (19) |
|  | Economy and law | 10 | 3 (4) | 7 (23) |
|  | Other | 19 | 5 (7) | 14 (45) |
| **Current semester (sum), mean (SD)** | | 4.55 (4.00) | 3.45 (3.42) | 7.03 (4.15) |
| **Employment, n (%)** | |  |  |  |
|  | Unemployed | 44 | 38 (55) | 6 (19) |
|  | Full-time | 6 | 1 (1) | 5 (16) |
|  | Part-time | 30 | 16 (23) | 14 (45) |
|  | Short time | 13 | 9 (13) | 4 (13) |
|  | Other | 2 | 2 (3) | 0 (0) |

^a^Percentage omitted due to redundancy.

^b^Missing demographic data due to unassignable data from the initial survey to the data from the smartphone study procedure.
