# Supplementary material for: Digital Stress Induction in Daily Life Using the Salzburg Mobile Stress Induction (SMSI): Development and Ambulatory Evaluation Study
Source: J Med Internet Res. 2025 Sep 18;27:e75785. doi: 10.2196/75785 (PMC12491893; doi:10.2196/75785)
Supplement: Multimedia Appendix 7 [file jmir_v27i1e75785_app7.doc]

## Multimedia Appendix 7

**Table S1.** Table of the two-way repeated measures ANOVA and contrast statistics for ***negative affect*** of the stress-inducing tests (Matrices test [MT], Cube Net test [CN], Arithmetic test [AR], Number Series test [NS], Word Scramble test [WS], and Word Pair test [WP]) against the Caesar Cipher control test (CC (CT)) of the Salzburg Mobile Stress Induction (***test***) from baseline (t0) to after the first task block (t1) and after the second task block assessments (t2; ***time***) controlling for ***sample*** (local university, n=69, and crowdsourcing subsample, n=31) as between-subject factor (N=100). Greenhouse-Geisser corrected degrees of freedom were used in the within-subjects effect statistics. η_p_²: partial eta squared.

| Within-subject effects | | *F* test (*df*) | | | | | | *P* value | | | η_p_² | |
| --- | --- | --- | --- | --- | --- | --- | --- | --- | --- | --- | --- | --- |
| Test | | 3.49 (5.50, 539.00) | | | | | | *.003*^a^ | | | 0.03 | |
| Test×sample | | 0.64 (5.50, 539.00) | | | | | | .682 | | | 0.01 | |
| Time | | 98.81 (1.18, 115.59) | | | | | | *<.001* | | | 0.50 | |
| Time×sample | | 0.88 (1.18, 115.59) | | | | | | .366 | | | 0.01 | |
| Test×time | | 6.06 (9.05, 886.46) | | | | | | *<.001* | | | 0.07 | |
| Test×time×sample | | 0.79 (9.05, 886.46) | | | | | | .627 | | | 0.01 | |
| CC (CT) vs. | t0 vs. t1 | | | | | | t0 vs. t2 | | | | | |
|  | *F* test  (1, 99) | | | *P* value | η_p_² | | *F* test  (1, 99) | | *P* value | | | η_p_² |
| MT | 20.47 | | | *<.001* | 0.17 | | 31.30 | | *<.001* | | | 0.24 |
| CN | 12.40 | | | *<.001* | 0.11 | | 8.20 | | *.005* | | | 0.08 |
| AR | 19.84 | | | *<.001* | 0.17 | | 18.59 | | *<.001* | | | 0.16 |
| NS | 19.43 | | | *<.001* | 0.17 | | 28.78 | | *<.001* | | | 0.23 |
| WS | 25.37 | | | *<.001* | 0.21 | | 27.71 | | *<.001* | | | 0.22 |
| WP | 13.44 | | | *<.001* | 0.12 | | 14.08 | | *<.001* | | | 0.13 |
| Between-Subject Effect | | | *F* test (*df*) | | | *P* value | | | | η_p_² | | |
| Sample | | | 0.01 (1, 98) | | | .934 | | | | 0.00 | | |

^a^Italics emphasize significance.

**Table S2.** Table of the two-way repeated measures ANOVA and contrast statistics for ***negative affect*** of the stress-inducing tests (Matrices test [MT], Cube Net test [CN], Arithmetic test [AR], Number Series test [NS], Word Scramble test [WS], and Word Pair test [WP]) against the Caesar Cipher control test (CC (CT)) of the Salzburg Mobile Stress Induction (***test***) from baseline (t0) to after the first task block (t1) and after the second task block assessments (t2; ***time***) controlling for ***gender*** (60/96, women and 34/96, men) as between-subject factor (N=96). Data from 5 participants were missing due to unassignable data from the initial survey to the data from the smartphone study procedure and 1 participant identified as non-binary and was excluded from the analysis. Greenhouse-Geisser corrected degrees of freedom were used in the within-subjects effect statistics. η_p_²: partial eta squared.

| Within-subject effects | | *F* test (*df*) | | | | *P* value | | | | η_p_² | |
| --- | --- | --- | --- | --- | --- | --- | --- | --- | --- | --- | --- |
| Test | | 3.98 (5.51, 507.12) | | | | *<.001* | | | | 0.04 | |
| Test×gender | | 0.65 (5.51, 507.12) | | | | .679 | | | | 0.01 | |
| Time | | 95.48 (1.18, 108.37) | | | | *<.001* | | | | 0.51 | |
| Time×gender | | 0.08 (1.18, 108.37) | | | | .819 | | | | 0.00 | |
| Test×time | | 6.15 (9.18, 844.55) | | | | *<.001* | | | | 0.06 | |
| Test×time×gender | | 1.08 (9.18, 844.55) | | | | .372 | | | | 0.01 | |
| CC (CT) vs. | t0 vs. t1 | | | | | | t0 vs. t2 | | | | |
|  | *F* test  (1, 92) | | *P* value | η_p_² | | | *F* test  (1, 92) | *P* value | | | η_p_² |
| MT | 24.14 | | *<.001* | 0.22 | | | 39.39 | *<.001* | | | 0.30 |
| CN | 12.43 | | *<.001* | 0.12 | | | 7.45 | *.008* | | | 0.08 |
| AR | 19.57 | | *<.001* | 0.18 | | | 18.45 | *<.001* | | | 0.17 |
| NS | 16.54 | | *<.001* | 0.15 | | | 27.12 | *<.001* | | | 0.23 |
| WS | 28.59 | | *<.001* | 0.24 | | | 28.42 | *<.001* | | | 0.24 |
| WP | 11.09 | | *.001* | 0.11 | | | 14.38 | *<.001* | | | 0.14 |
| Between-subject effect | | *F* test (*df*) | | | *P* value | | | | η_p_² | | |
| Gender | | 0.15 (1, 92) | | | .903 | | | | 0.00 | | |

^a^Italics emphasize significance.

**Table S3.** Table of the two-way repeated measures ANOVA and contrast statistics for ***negative affect*** of the stress-inducing tests (Matrices test [MT], Cube Net test [CN], Arithmetic test [AR], Number Series test [NS], Word Scramble test [WS], and Word Pair test [WP]) against the Caesar Cipher control test (CC (CT)) of the Salzburg Mobile Stress Induction (***test***) from baseline (t0) to after the first task block (t1) and after the second task block assessments (t2; ***time***) controlling for ***employment*** (employed, n=51, and unemployed, n=44) as between-subject factor (N=95). Greenhouse-Geisser corrected degrees of freedom were used in the within-subjects effect statistics. η_p_²: partial eta squared.

| Within-subject effects | | *F* test (*df*) | | | | *P* value | | | | η_p_² | |
| --- | --- | --- | --- | --- | --- | --- | --- | --- | --- | --- | --- |
| Test | | 4.93 (5.51, 512.76) | | | | *<.001*^a^ | | | | 0.05 | |
| Test×employment | | 1.18 (5.51, 512.76) | | | | .315 | | | | 0.01 | |
| Time | | 106.65 (1.18, 109.95) | | | | *<.001* | | | | 0.53 | |
| Time×employment | | 1.85 (1.18, 109.95) | | | | .176 | | | | 0.02 | |
| Test×time | | 7.22 (9.05, 841.97) | | | | *<.001* | | | | 0.07 | |
| Test×time×employment | | 0.73 (9.05, 841.97) | | | | .682 | | | | 0.01 | |
| CC (CT) vs. | t0 vs. t1 | | | | | | t0 vs. t2 | | | | |
|  | *F* test  (1, 99) | | *P* value | η_p_² | | | *F* test  (1, 99) | *P* value | | | η_p_² |
| MT | 26.70 | | *<.001* | 0.22 | | | 40.50 | *<.001* | | | 0.30 |
| CN | 15.78 | | *<.001* | 0.15 | | | 9.53 | *.003* | | | 0.09 |
| AR | 17.09 | | *<.001* | 0.16 | | | 29.66 | *<.001* | | | 0.24 |
| NS | 25.59 | | *<.001* | 0.22 | | | 22.61 | *<.001* | | | 0.20 |
| WS | 32.41 | | *<.001* | 0.26 | | | 33.39 | *<.001* | | | 0.26 |
| WP | 14.34 | | *<.001* | 0.13 | | | 16.45 | *<.001* | | | 0.15 |
| Between-subject effect | | *F* test (*df*) | | | *P* value | | | | η_p_² | | |
| Employment | | 0.96 (1, 93) | | | .757 | | | | 0.00 | | |

^a^Italics emphasize significance.
