# Supplementary material for: Digital Stress Induction in Daily Life Using the Salzburg Mobile Stress Induction (SMSI): Development and Ambulatory Evaluation Study
Source: J Med Internet Res. 2025 Sep 18;27:e75785. doi: 10.2196/75785 (PMC12491893; doi:10.2196/75785)
Supplement: Multimedia Appendix 8 [file jmir_v27i1e75785_app8.doc]

## Multimedia Appendix 8

**Table S1.** Table of the two-way repeated measures ANOVA and contrast statistics for ***positive affect*** of the stress-inducing tests (Matrices test [MT], Cube Net test [CN], Arithmetic test [AR], Number Series test [NS], Word Scramble test [WS], and Word Pair test [WP]) against the Caesar Cipher control test (CC (CT)) of the Salzburg Mobile Stress Induction (***test***) from baseline (t0) to after the first task block (t1) and after the second task block assessments (t2; ***time***) controlling for ***sample*** (local university, n=69, and crowdsourcing subsample, n=31) as between-subject factor (N=100). Greenhouse-Geisser corrected degrees of freedom were used in the within-subjects effect statistics. η_p_²=partial eta squared.

| Within-subject effects | | *F* test (*df*) | | | | *P* value | | | | η_p_² | |
| --- | --- | --- | --- | --- | --- | --- | --- | --- | --- | --- | --- |
| Test | | 3.45 (5.21, 510.93) | | | | *.004*^a^ | | | | 0.03 | |
| Test×sample | | 1.78 (5.21, 510.93) | | | | .112 | | | | 0.02 | |
| Time | | 6.34 (1.27, 123.92) | | | | *.008* | | | | 0.06 | |
| Time×sample | | 3.61 (1.27, 123.92) | | | | .050 | | | | 0.04 | |
| Test×time | | 7.53 (8.43, 826.13) | | | | *<.001* | | | | 0.07 | |
| Test×time×sample | | 1.39 (8.43, 826.13) | | | | .194 | | | | 0.01 | |
| CC (CT) vs. | t0 vs. t1 | | | | | | t0 vs. t2 | | | | |
|  | *F* test  (1, 99) | | *P* value | η_p_² | | | *F* test  (1, 99) | *P* value | | | η_p_² |
| MT | 15.86 | | *<.001* | 0.14 | | | 37.51 | *<.001* | | | 0.28 |
| CN | 15.80 | | *<.001* | 0.14 | | | 20.14 | *<.001* | | | 0.17 |
| AR | 20.25 | | *<.001* | 0.17 | | | 38.08 | *<.001* | | | 0.28 |
| NS | 13.96 | | *<.001* | 0.13 | | | 15.07 | *<.001* | | | 0.13 |
| WS | 34.15 | | *<.001* | 0.26 | | | 42.76 | *<.001* | | | 0.30 |
| WP | 15.14 | | *<.001* | 0.13 | | | 18.71 | *<.001* | | | 0.16 |
| Between-subject effect | | *F* test (*df*) | | | *P* value | | | | η_p_² | | |
| Sample | | 25.79 (1, 98) | | | *<.001* | | | | 0.21 | | |

^a^Italics emphasize significance.

**Table S2.** Table of Mean and SD scores of ***positive affect*** for each ***test*** (Matrices test [MT], Cube Net test [CN], Arithmetic test [AR], Number Series test [NS], Word Scramble test [WS], Word Pair test [WP], and the Caesar Cipher control test [CC (CT)]) of the Salzburg Mobile Stress Induction and measurement ***time*** (t0, baseline; t1, after the first task block, t2, after the second task block) displayed for the ***total*** sample, the ***local university*** and the ***crowdsourcing*** subsample.

|  | Total sample (N=100) | | Local university subsample (n=69) | | Crowdsourcing subsample (n=31) | |
| --- | --- | --- | --- | --- | --- | --- |
|  | Mean | SD | Mean | SD | Mean | SD |
| **MT** | | | | | | |
| t0 | 53.60 | 23.97 | 47.71 | 22.67 | 66.70 | 21.78 |
| t1 | 52.83 | 23.82 | 47.16 | 21.45 | 65.46 | 24.29 |
| t2 | 48.40 | 23.40 | 43.99 | 21.24 | 58.20 | 25.29 |
| **CN** | | | | | | |
| t0 | 48.76 | 22.10 | 41.49 | 20.38 | 64.94 | 16.71 |
| t1 | 47.79 | 22.08 | 40.95 | 20.79 | 62.99 | 16.83 |
| t2 | 46.91 | 22.66 | 40.73 | 21.08 | 60.67 | 20.08 |
| **AR** | | | | | | |
| t0 | 49.79 | 23.25 | 42.22 | 21.71 | 66.65 | 17.04 |
| t1 | 49.59 | 23.72 | 44.84 | 23.03 | 60.14 | 22.08 |
| t2 | 46.59 | 23.26 | 41.70 | 22.78 | 57.46 | 20.78 |
| **NS** | | | | | | |
| t0 | 52.43 | 22.79 | 46.60 | 21.69 | 65.41 | 19.89 |
| t1 | 51.78 | 23.55 | 46.98 | 23.52 | 62.47 | 20.14 |
| t2 | 50.78 | 24.32 | 45.31 | 24.02 | 62.95 | 20.54 |
| **WS** | | | | | | |
| t0 | 54.32 | 24.82 | 46.63 | 23.51 | 71.45 | 18.48 |
| t1 | 50.88 | 23.58 | 44.31 | 22.47 | 65.50 | 19.23 |
| t2 | 49.60 | 24.23 | 43.22 | 23.01 | 63.81 | 20.87 |
| **WP** | | | | | | |
| t0 | 56.29 | 21.61 | 51.27 | 21.59 | 67.45 | 17.27 |
| t1 | 56.11 | 20.10 | 51.70 | 20.45 | 65.93 | 15.52 |
| t2 | 53.38 | 22.01 | 47.96 | 21.87 | 65.44 | 17.26 |
| **CC (CT)** | | | | | | |
| t0 | 51.61 | 20.48 | 47.59 | 19.19 | 60.57 | 20.71 |
| t1 | 58.11 | 20.37 | 54.75 | 21.75 | 65.58 | 14.57 |
| t2 | 58.81 | 20.86 | 55.65 | 21.26 | 65.86 | 18.37 |

**Table S3.** Table of the two-way repeated measures ANOVA and contrast statistics for ***positive affect*** of the stress-inducing tests (Matrices test [MT], Cube Net test [CN], Arithmetic test [AR], Number Series test [NS], Word Scramble test [WS], and Word Pair test [WP]) against the Caesar Cipher control test (CC (CT)) of the Salzburg Mobile Stress Induction (***test***) from baseline (t0) to after the first task block (t1) and after the second task block assessments (t2; ***time***) controlling for ***gender*** (60/100, women and 34/100, men) as between-subject factor (N=100). Data from 5 participants were missing due to unassignable data from the initial survey to the data from the smartphone study procedure and 1 participant identified as non-binary and was excluded from the analysis. Greenhouse-Geisser corrected degrees of freedom were used in the within-subjects effect statistics. η_p_²=partial eta squared.

| Within-subject effects | | *F* test (*df*) | | | | *P* value | | | | η_p_² | |
| --- | --- | --- | --- | --- | --- | --- | --- | --- | --- | --- | --- |
| Test | | 4.61 (5.33, 490.29) | | | | *<.001*^a^ | | | | 0.05 | |
| Test×gender | | 1.47 (5.33, 490.29) | | | | .195 | | | | 0.02 | |
| Time | | 3.84 (1.26, 115.46) | | | | *.023* | | | | 0.04 | |
| Time×gender | | 0.24 (1.26, 115.46) | | | | .678 | | | | 0.00 | |
| Test×time | | 7.09 (8.49, 781.19) | | | | *<.001* | | | | 0.07 | |
| Test×time×gender | | 0.43 (8.49, 781.19) | | | | .913 | | | | 0.01 | |
| CC (CT) vs. | t0 vs. t1 | | | | | | t0 vs. t2 | | | | |
|  | *F* test  (1, 92) | | *P* value | η_p_² | | | *F* test  (1, 92) | *P* value | | | η_p_² |
| MT | 16.34 | | *<.001* | 0.15 | | | 33.95 | *<.001* | | | 0.27 |
| CN | 16.07 | | *<.001* | 0.15 | | | 17.01 | *<.001* | | | 0.16 |
| AR | 14.41 | | *<.001* | 0.14 | | | 29.70 | *<.001* | | | 0.24 |
| NS | 12.04 | | *<.001* | 0.12 | | | 15.61 | *<.001* | | | 0.15 |
| WS | 30.86 | | *<.001* | 0.25 | | | 37.10 | *<.001* | | | 0.29 |
| WP | 17.02 | | *<.001* | 0.16 | | | 25.37 | *<.001* | | | 0.22 |
| Between-subject effect | | *F* test (*df*) | | | *P* value | | | | η_p_² | | |
| Gender | | 1.79 (1, 92) | | | .184 | | | | .02 | | |

^a^Italics emphasize significance.

**Table S4.** Table of the two-way repeated measures ANOVA and contrast statistics for ***positive affect*** of the stress-inducing tests (Matrices test [MT], Cube Net test [CN], Arithmetic test [AR], Number Series test [NS], Word Scramble test [WS], and Word Pair test [WP]) against the Caesar Cipher control test (CC (CT)) of the Salzburg Mobile Stress Induction (***test***) from baseline (t0) to after the first task block (t1) and after the second task block assessments (t2; ***time***) controlling for ***employment*** (employed, n=51, and unemployed, n=44) as between-subject factor (N=95). Greenhouse-Geisser corrected degrees of freedom were used in the within-subjects effect statistics. η_p_²=partial eta squared.

| Within-subject effects | | *F* test (*df*) | | | | *P* value | | | | η_p_² | |
| --- | --- | --- | --- | --- | --- | --- | --- | --- | --- | --- | --- |
| Test | | 6.00 (5.40, 502.27) | | | | *<.001*^a^ | | | | 0.06 | |
| Test×employment | | 1.04 (5.40, 502.27) | | | | .397 | | | | 0.01 | |
| Time | | 4.42 (1.26, 116.87) | | | | *.029* | | | | 0.05 | |
| Time×employment | | 0.20 (1.26, 116.87) | | | | .710 | | | | 0.00 | |
| Test×time | | 7.86 (8.60, 799.82) | | | | *<.001* | | | | 0.08 | |
| Test×time×employment | | 0.35 (8.60, 799.82) | | | | .956 | | | | 0.00 | |
| CC (CT) vs. | t0 vs. t1 | | | | | | t0 vs. t2 | | | | |
|  | *F* test  (1, 99) | | *P* value | η_p_² | | | *F* test  (1, 99) | *P* value | | | η_p_² |
| MT | 17.66 | | *<.001* | 0.16 | | | 37.50 | *<.001* | | | 0.29 |
| CN | 17.03 | | *<.001* | 0.16 | | | 20.15 | *<.001* | | | 0.18 |
| AR | 13.70 | | *<.001* | 0.13 | | | 32.90 | *<.001* | | | 0.26 |
| NS | 14.14 | | *<.001* | 0.13 | | | 18.46 | *<.001* | | | 0.17 |
| WS | 34.40 | | *<.001* | 0.27 | | | 43.25 | *<.001* | | | 0.32 |
| WP | 19.66 | | *<.001* | 0.18 | | | 27.98 | *<.001* | | | 0.23 |
| Between-subject effect | | *F* test (*df*) | | | *P* value | | | | η_p_² | | |
| Employment | | 10.04 (1, 93) | | | *.002* | | | | 0.10 | | |

^a^Italics emphasize significance.

**Table S5.** Table of Mean and SD scores of ***positive affect*** for each ***test*** (Matrices test [MT], Cube Net test [CN], Arithmetic test [AR], Number Series test [NS], Word Scramble test [WS], Word Pair test [WP], and the Caesar Cipher control test [CC (CT)]) of the Salzburg Mobile Stress Induction and measurement ***time*** (t0, baseline; t1, after the first task block, t2, after the second task block) displayed for the ***total*** sample, ***employed*** and ***unemployed*** participants.

|  | Total sample (N=95) | | Employed (n=51) | | Unemployed (n=44) | |
| --- | --- | --- | --- | --- | --- | --- |
|  | Mean | SD | Mean | SD | Mean | SD |
| **MT** | | | | | | |
| t0 | 53.50 | 23.85 | 58.49 | 3.27 | 47.71 | 3.52 |
| t1 | 52.71 | 23.61 | 56.91 | 3.26 | 47.85 | 3.51 |
| t2 | 48.61 | 22.80 | 52.77 | 3.15 | 43.78 | 3.39 |
| **CN** | | | | | | |
| t0 | 48.27 | 21.49 | 54.55 | 2.87 | 41.00 | 3.09 |
| t1 | 47.53 | 21.73 | 54.61 | 2.86 | 39.33 | 3.08 |
| t2 | 47.05 | 22.44 | 53.97 | 2.98 | 39.03 | 3.21 |
| **AR** | | | | | | |
| t0 | 49.63 | 23.04 | 54.06 | 3.17 | 44.49 | 3.42 |
| t1 | 49.52 | 23.51 | 53.06 | 3.27 | 45.43 | 3.52 |
| t2 | 46.41 | 22.99 | 50.93 | 3.16 | 41.17 | 3.40 |
| **NS** | | | | | | |
| t0 | 52.20 | 22.42 | 56.05 | 3.10 | 47.75 | 3.34 |
| t1 | 52.15 | 23.33 | 56.65 | 3.21 | 46.92 | 3.46 |
| t2 | 50.62 | 24.60 | 55.60 | 3.38 | 44.86 | 3.64 |
| **WS** | | | | | | |
| t0 | 53.94 | 24.78 | 60.83 | 3.33 | 45.95 | 3.58 |
| t1 | 50.54 | 23.39 | 57.36 | 3.12 | 42.62 | 3.36 |
| t2 | 49.21 | 23.99 | 56.51 | 3.19 | 40.76 | 3.43 |
| **WP** | | | | | | |
| t0 | 56.71 | 21.25 | 61.32 | 2.91 | 51.35 | 3.13 |
| t1 | 56.01 | 20.27 | 61.18 | 2.74 | 50.01 | 2.95 |
| t2 | 53.08 | 21.60 | 58.74 | 2.92 | 46.52 | 3.14 |
| **CC (CT)** | | | | | | |
| t0 | 51.89 | 20.23 | 56.00 | 2.78 | 47.12 | 2.99 |
| t1 | 58.21 | 20.23 | 61.94 | 2.79 | 53.90 | 3.00 |
| t2 | 59.06 | 20.72 | 63.13 | 2.85 | 54.34 | 3.07 |
